# Shotgun metagenomic sequencing from Manao-Pee cave, Thailand, reveals insight into the microbial community structure and its metabolic potential

**DOI:** 10.1186/s12866-019-1521-8

**Published:** 2019-06-27

**Authors:** Apirak Wiseschart, Wuttichai Mhuantong, Sithichoke Tangphatsornruang, Duriya Chantasingh, Kusol Pootanakit

**Affiliations:** 1 Institute of Molecular Biosciences, Mahidol University, Salaya Campus, Phuttamonthon 4 Rd, Salaya, Nakhon Pathom, 73170 Thailand; 2 National Center for Genetic Engineering and Biotechnology (BIOTEC), 133 Thailand Science Park, Paholyothin Rd, Klong 1, Klongluang, Pathumthani 12120 Thailand

**Keywords:** Manao-Pee cave, Culture-independent approach, Shotgun metagenomic sequencing, Metabolic potential analysis, KEGG pathways

## Abstract

**Background:**

Due to the cave oligotrophic environment, this habitat presents a challenge for microorganisms to colonize and thrive. However, it has been well documented that microorganisms play important roles in cave development. Survival of microbes in this unique habitat likely involves a broad range of adaptive capabilities. Recently, cave microbiomes all over the world are of great scientific interest. However, the majority of investigations focused mostly on small subunit ribosomal RNA (16S rRNA) gene, leaving the ecological role of the microbial community largely unknown. Here, we are particularly interested in exploring the taxonomic composition and metabolic potential of microorganisms in soil from Manao-Pee cave, a subterranean limestone cave in the western part of Thailand, by using high-throughput shotgun metagenomic sequencing.

**Results:**

From taxonomic composition analysis using ribosomal RNA genes (rRNA), the results confirmed that *Actinobacteria* (51.2%) and *Gammaproteobacteria* (24.4%) were the dominant bacterial groups in the cave soil community. Metabolic potential analysis, based on six functional modules of the Kyoto Encyclopedia of Genes and Genomes (KEGG) database, revealed that functional genes involved in microbial metabolisms are highly represented in this community (40.6%). To better understand how microbes thrive under unfavorable cave condition, we focused on microbial energy metabolism. The results showed that microbial genes involved in oxidative phosphorylation were the most dominant (28.8%) in Manao-Pee cave, and were followed by methane metabolism (20.5%), carbon fixation (16.0%), nitrogen metabolism (14.7%), and sulfur metabolism (6.3%). In addition, microbial genes involved in xenobiotic biodegradation (26 pathways) and in production of secondary metabolites (27 pathways) were also identified.

**Conclusion:**

In addition to providing information on microbial diversity, we also gained insights into microbial adaptations and survival strategies under cave conditions. Based on rRNA genes, the results revealed that bacteria belonging to the *Actinobacteria* and *Gammaproteobacteria* were the most abundant in this community. From metabolic potential analysis, energy and nutrient sources that sustain diverse microbial population in this community might be atmospheric gases (methane, carbon dioxide, nitrogen), inorganic sulfur, and xenobiotic compounds. In addition, the presence of biosynthetic pathways of secondary metabolites suggested that they might play important ecological roles in the cave microbiome.

**Electronic supplementary material:**

The online version of this article (10.1186/s12866-019-1521-8) contains supplementary material, which is available to authorized users.

## Background

Recent high-throughput sequencing technologies are robust and economical for microbial ecology study. 16S rRNA gene is usually directly amplified from extracted metagenomic DNA of environmental samples for the exploration of prokaryotic diversity [1, 2]. However, it is well-known that PCR-based studies have several limitations [3]. For instance, variability in PCR amplification efficiency, chimera formation (hybrid products between multiple parent sequences), and potential PCR primers bias [1, 4, 5]. As high-throughput sequencing technologies typically generate short reads, only a single or a combination of neighbouring hypervariable regions of 16S rRNA gene are normally used in microbial diversity analysis. It is worth noting that different genes as well as different regions of the same gene usually have different accuracy and coverage for their operational taxonomic unit (OTU) abundance estimation [6–8]. Primer selection is thus very important in a PCR-based microbial ecology study. In addition, as this technique gives only the relative abundance information, the ecological roles of microbial communities thus largely remained unknown [8, 9].

As one of the main objectives in the study of microbial ecology is to establish a relationship between taxonomic distribution, i.e. who are there, and their ecological functions, i.e. what are they capable of doing, of complex microbial communities [10]. Thus, in the last few years, several bioinformatic approaches have been used to predict metabolic potential of the microbial community using information based solely on the obtained 16S rRNA gene sequences [11–13]. As this prediction is always based on the marker gene amplification, the accuracy of such an approach is also dependent on how reliable is the PCR technique employed. In addition, availability of nutrients and chemicals in ecological niches also influence metabolic activity of microorganisms [14]; consequently, their ecological activities are very complex to predict. Last but not least, functional prediction based on what we know about closely related species is not always true as even those closely related microorganisms frequently have different important functional genes [15]. Therefore, it would be better to gain a deeper insight into community’s functional capabilities from unamplified environmental DNA (eDNA).

Shotgun metagenomic DNA sequencing does not rely on PCR amplification using gene-targeted primers; thus, it is a powerful technique in the field of molecular microbial ecology [8]. Because of the technical differences, amplicon sequencing and metagenomic shotgun sequencing may not give identical results. However, it has been reported that metagenomic shotgun sequencing provides a more robust and reliable assessment of the microbial diversity [3, 16]. Additionally, it not only provides a direct assessment of the microbial profile but also gives valuable information on metabolic potential of the microbial community [3, 8, 17]. However, one of the drawbacks to this approach is the incomplete gene annotations as only limited numbers of bacterial genomes are available [6]. Also, when compared to amplicon sequencing, the cost of shotgun sequencing is more expensive and requires more extensive data analysis [3, 8, 16]. Even with these limitations, it is still a promising molecular technique to close the gap between community structure and functional capability, contributing to a better understanding of how microbes thrive and adapt under natural conditions especially in less explored habitats. Here, we used Ion Torrent PGM to obtain the eDNA reads. Eventhough this platform gives higher indel errors especially in the homopolymer regions when compared to others [18], it nevertheless achieved comparable results when compared to the Illumina MiSeq datasets for functional categorization of assembled shotgun sequences [19].

Cave microbiomes are one of the least studied biomes [20]. This may be because of restrictions to avoid damage to natural resources, or that cave sampling is not as easy when compared to surface habitats. In the cave ecosystem, beyond the twilight zone, photosynthesis does not occur. Consequently, oligotrophic conditions are always found deep inside the caves. Even with this nutrient-limited condition, they are by no means barren or lifeless. A wide spectrum of microorganisms can thrive in the cave environment [21]. It has been reported that microorganisms play important ecological roles in cave development via direct or indirect activities [22, 23]. Recently, microbial diversity has been investigated in caves around the world. Based on 16S rRNA gene sequencing, mounting evidence indicates that *Actinobacteria* and *Proteobacteria* are the most ubiquitous bacteria phyla in cave ecosystems [21, 24]. As opposed to microbial profiling data, information on metabolic potential of the cave microbiome is very limited. To the best of our knowledge, only a few studies have shown metabolic capabilities of cave microorganisms. For instance, results from the metabolic potential analysis of microorganisms on carbonate speleothem surfaces of Kartchner Caverns, USA, revealed that the prokaryotic community genetically adapted to low-nutrient conditions by using alternative non-photosynthetic primary production strategies (e.g. CO_2_ fixation, nitrogen metabolism) [25]. Yet another study investigated microbial communities embedded in a secondary mineral deposit from Tjuv-Ante’s cave identified microbial genes related to iron and sulfur metabolisms [26].

However, to date no such study has been conducted on cave soil sediments. Analysis of microbial metabolic potential of this community might provide opportunities to expand our understanding about survival strategies of cave microorganisms. Therefore, the present study aimed to explore microbial community structure and also metabolic potential of the soil community from a subterranean limestone cave of Khao Wang Khamen. As far as we know, this study is the first shotgun metagenomic sequencing of cave microbiome in Thailand. The work presented fills a much needed knowledge gap regarding microbial community structure and on how cave-dwelling microbes thrive under energetically unfavorable and nutrient-limited conditions.

## Methods

### Site description and sampling

Manao-Pee cave is part of the extensive Khao Wang Khamen karst system. It is located in Kanchanaburi province, in the western part of Thailand. The cave is off-limit to tourists and the public as it is under the protection of The Royal Thai Armed Forces Development Command. Within the cave, a wide diversity of white to brown-orange calcite speleothems (secondary mineral deposits) are present. In the current study, we are particularly interested in the cave soil since we wanted to know how microorganisms survive under nutrient-limited cave condition. When compared to the soil outside the cave, the cave soil is more brownish and sandy. In terms of geochemical composition, our previous study has shown that copper (Cu), iron (Fe), manganese (Mn), and zinc (Zn) were found at higher concentrations in soil samples from the cave [27]. Conversely, organic matter, total carbon, total nitrogen, and total hydrogen were found to be at lower concentrations when compared to soil samples outside the cave. For the present study, soil samples were collected from 5 different locations inside the dark zone of the cave, about 20–50 m from the cave entrance. All soil samples were kept on ice before returning to the laboratory and immediately stored at − 20 °C until processed.

### Total environmental DNA extraction and purification

Total environmental DNA (eDNA) was directly extracted from a composite soil sample as described previously [27]. Specifically, 5 g of composite soil was mixed with 13.5 ml of DNA extraction buffer (100 mM Tris-HCl (pH 8.0), 100 mM sodium EDTA (pH 8.0), 100 mM sodium phosphate (pH 8.0), 1.5 M NaCl, 1% (w/v) cetyltrimethylammonium bromide (CTAB)) and 100 μl of proteinase K (10 mg/ml). The mixture was vigorously shaken at 250 rpm, 37 °C for 30 min. Then, 1.5 ml of 20% (w/v) SDS was added to the mixture. It was further incubated at 65 °C for 2 h. During this time, the mixture was gently mixed every 15–20 min. After 2 h, the mixture was centrifuged at 6000×*g* for 10 min at room temperature. The supernatant was transferred to new 50 ml centrifuge tube, the soil pellet was re-extracted by adding 4.5 ml of DNA extraction buffer and 0.5 ml of 20% (w/v) SDS. It was further incubated at 65 °C for 15 min. After that, the supernatant was collected as described above. All supernatants were then combined and mixed with equal volume of chloroform: isoamylalcohol (24:1 (v/v)). The upper phase was collected to new tube after centrifugation at 6000×*g* for 10 min at 4 °C. eDNA was precipitated with 0.7 volumes of isopropanol and incubated for 1 h at room temperature. The precipitated eDNA was pelleted by centrifugation at 16,000×*g* for 20 min at 4 °C. 300 μl of ice-cold 70% ethanol (v/v) was added to the DNA pellet and centrifuged again at 16,000×*g* for 10 min at 4 °C. Then, eDNA pellet was air-dried at room temperature and resuspended in 100 μl of sterilized MiliQ water. Finally, the extracted eDNA was purified by using electroelution technique.

### Purification of environmental DNA by electroelution technique

Extracted eDNA was purified by electroelution technique [28], with slight modifications. Specifically, crude eDNA was separated through 1% agarose gel in 0.5X TBE buffer with constant voltage of 100 v. After 1 h, any remaining DNA that did not migrate into the agarose gel was washed out of the loading wells, and the buffer was replaced. The voltage was then reduced to 20 v, and the gel was run for 5 h. Then, a slice of agarose gel containing high molecular weight eDNA was then cut out with a sharp scalpel. The excised gel was then put into a dialysis bag (Spectra/Por, USA) filled with 0.25x TBE buffer. Next, the bag was immersed in a shallow layer of 0.25x TBE buffer in a horizontal electrophoresis tank in an orientation that was inline with the electrodes, with the current at 100 v for 90 min. The polarity of electric current was then reversed for 1 min to release any eDNA sticking to the wall of dialysis bag. The buffer with eluted eDNA was transferred into a 15 ml tube. One volume of chloroform:isoamylalcohol (24:1 (v/v)) was added and mixed before centrifugation at 6000×*g* for 10 min. The upper phase was transferred to a clean 15 ml tube. Eluted eDNA was then precipitated by adding 1/10 volume of 3 M sodium acetate (pH 5.2) and 0.7 volume of isopropanol. After 1 h of incubation at room temperature, eluted eDNA was pelleted by centrifugation at 13,000×*g* for 20 min. After washing with 70% ethanol (v/v), eDNA pellet was air-dried at room temperature and resuspended in 50 μl of sterilized MiliQ water.

### Shotgun metagenomic sequencing and metabolic potential analysis

Purified eDNA was sequenced using Ion Proton sequencing system (Life Technologies, USA) following the manufacturer’s protocol. The metagenomic sequence reads obtained are available at the NCBI Sequence Read Archive (https://www.ncbi.nlm.nih.gov/sra) with the accession number PRJNA485054. The quality of sequencing datasets were initially assessed using FastQC software [29]. Sequences with low Phred quality score (< 20) and shorter than 100 base pairs in length were filtered out. The remaining sequences were translated into amino acid sequences for their protein-coding regions through the MetaGeneMark program [30]. The functional annotation of metagenomic sequences was performed by the Reduced Alphabet based Protein similarity Search tool (RAPSEARCH2) [31] against the UniRef90 database [32]. The similarity search output from RAPSEARH was subsequently assigned for functional annotation using KEGG classification through Metagenome Analyzer (MEGAN) tool (version 6) [33]. Meanwhile, the remaining non-protein coding sequences were identified for rRNA sequences using BLASTN against the SILVA database [34]. All BLAST searches for rRNA genes were performed using the expected cutoff value of 1e^− 6^.

## Results

### Shotgun sequencing dataset

Purified eDNA was subjected to high-throughput Ion Proton sequencing. Approximately 19 million sequences with an average read length of 184 bp were obtained. After quality control, 17,353,239 (87.9%) sequence reads were suitable for further bioinformatic analysis. This number included both coding and non-coding sequences. Specifically, a total reads of 4,556,991 (26.3%) were of non-coding sequences. From this dataset, 46,567 reads contained rRNA genes (1.0%) that can be taxonomically assigned to cellular organisms. For coding sequences, a total reads of 12,796,248 (73.7%) was subjected to KEGG analysis for Manao-Pee’s metabolic potential.

### Microbial community structure in the Manao-Pee cave soil community through 16S rRNA gene

**Fig. 1:**
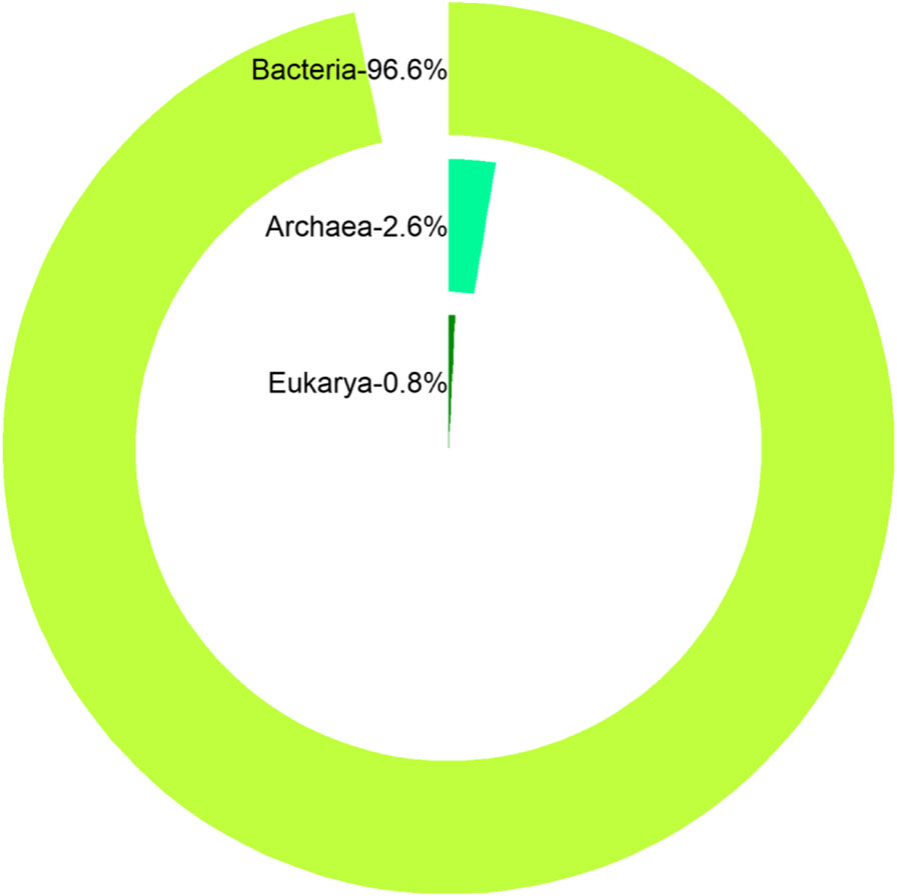
Distribution of organisms in soil community from the Manao-Pee cave. Percentage values represent the relative abundance of non-coding genes assigned to a particular domain

As expected most of the 46,567 rRNA genes identified can be phylogenetically assigned to the bacteria domain (96.6%). Only a small fraction were assigned to the archaeal (2.6%) and eukaryotic domains (0.8%) (Fig. 1).

**Fig. 2:**
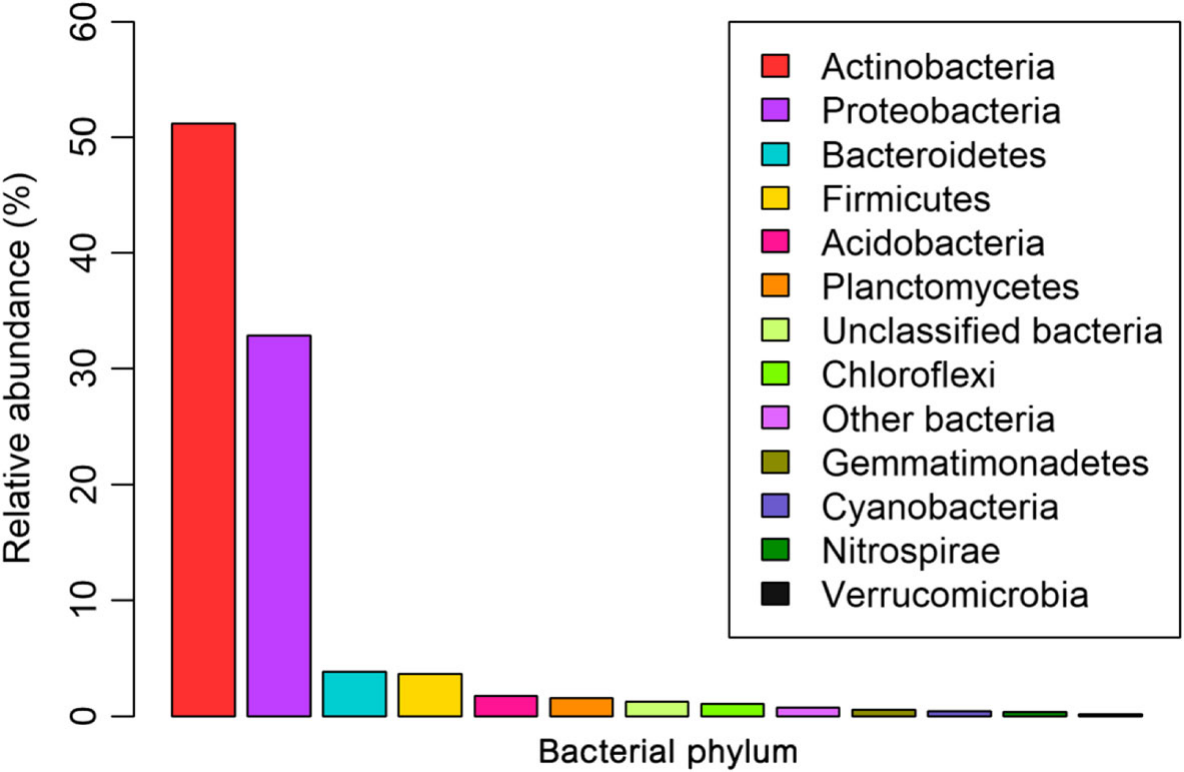
Bacterial diversity in soil community from the Manao-Pee cave. Percentage values represent the relative abundance of non-coding genes assigned to a particular phylum

*Actinobacteria* (51.2%) and *Proteobacteria* (32.9%) were the most abundant phyla in the cave soil community. Other bacterial phyla were also identified, but they were much less abundant, namely, *Bacteroidetes* (3.9%), *Fimicutes* (3.7%), *Acidobacteria* (1.8%), *Planctomycetes* (1.6%), *Chloroflexi* (1.1%), *Gemmatimonadetes* (0.6%), and *Cyanobacteria* (0.5%) (Fig. 2).

Among the *Actinobacteria,* 36 families were identified. The most abundant was *Pseudonocardiaceae* (21.1%), followed by *Nocardioidaceae* (17.8%), *Streptomycetaceae* (11.5%), *Mycobacteriaceae* (14.3%), *Micromonosporaceae* (8.2%), *Nocardiaceae* (5.0%), *Thermomonosporaceae* (3.3%), *Frankiaceae* (2.3%), *Propionibacteriaceae* (1.9%), *Glycomycetaceae* (1.4%), *Sporichthyaceae* (1.2%), *Intrasporangiaceae* (1.2%), and *Microbacteriaceae* (1.1%). The remaining 23 families have less than 1% (Additional file 1: Figure S1a). At the genus level, 117 genera were identified. *Mycobacterium* (15.1%) was the most abundant genus, followed by *Streptomyces* (11.9%), *Nocardioides* (9.4%), *Marmoricola* (4.7%), *Crossiella* (4.1%), *Amycolatopsis* (4.0%), *Nocardia* (3.1%), *Pseudonocardia* (2.8%), *Saccharopolyspora* (2.7%), *Actinoplanes* (2.1%), *Frankia* (2%), *Spirillospora* (1.8%), *Aeromicrobium* (1.8%), *Micromonospora* (1.5%), *Rhodococcus* (1.4%), *Salinispora* (1.4%), *Saccharomonospora* (1.3%), *Sporichthya* (1.2%), *Saccharothrix* (1.1%), *Stackebrandtia* (1.1%) and *Mumia* (1.0%). The remaining genera accounted for less than 1% (Additional file 1: Figure S1b).

*Proteobacteria* was the second most prevalent phylum in this community. Most of these sequences can be classified into the class *Gammaproteobacteria* (77.4%), followed by *Betaproteobacteria* (10.2%), *Alphaproteobacteria* (8.6%), *Deltaproteobacteria* (3.5%), and *Epsilonproteobacteria* (0.3%) (Additional file 2: Figure S2a). Amoung the 81 families identified, *Xanthomonadaceae* (43.9%) was the most abundant in this community, followed by *Burkholderiaceae* (7.9%)*, Ectothiorhodospiraceae* (6.9%), *Salinisphaeraceae* (5.0%), *Enterobacteriaceae* (4.1%), *Chromatiaceae* (3.7%), *Pseudomonadaceae* (3.2%), *Sphingomonadaceae* (1.7%), *Rhodospirillaceae* (1.6%), *Methylococcaceae* (1.6%), *Coxiellaceae* (1.5%), *Halomonadaceae* (1.3%), *Rhodobacteraceae* (1.2%), *Piscirickettsiaceae* (1.1%), and *Moraxellaceae* (1.0%). The other families accounted for less than 1% (Additional file 2: Figure S2b). Among 171 genera identified, *Burkholderia* (11.9%) was the most abundant genus in this community, followed by *Salinisphaera* (6.6%), *Lysobacter* (6.1%), *Xanthomonas* (5.5%), *Thioalkalivibrio* (5.1%), *Arenimonas* (3.9%), *Pseudomonas* (3.8%), *Pseudoxanthomonas* (2.9%), *Coxiella* (2.5%), *Sphingomonas* (2.2%), *Spiribacter* (2.0%), *Nitrococcus* (1.8%), *Thermomonas* (1.4%), *Rhizobium* (1.3%), *Cycloclasticus* (1.1%), *Nitrosococcus* (1.1%), *Methylobacter* (1.1%), *Halomonas* (1.1%), *Ectothiorhodospira* (1.0%), and *Salmonella* (1.0%). The remaining genera accounted for less than 1% (Additional file 2: Figure S2c).

*Thaumarchaeota* (90.7%) occupied the largest proportion in the archaeal domain. However, all reads assigned to this phylum could not be classified into deeper taxonomic level. *Euryarchaeota* (8.9%) was the second most dominant phylum, followed by *Crenarchaeota* (0.4%) (Additional file 3: Figure S3).

Among the eukaryotic organisms, the kingdom fungi (82.9%) was the most prevalent in this community with the division *Ascomycota* contributing 62.6%, followed by *Basidiomycota* (16.0%), *Mucoromycota* (2.4%), and *Zoopagomycota* (1.9%) (Additional file 4: Figure S4).

### Metabolic potential analysis of cave-dwelling microorganisms

**Fig. 3:**
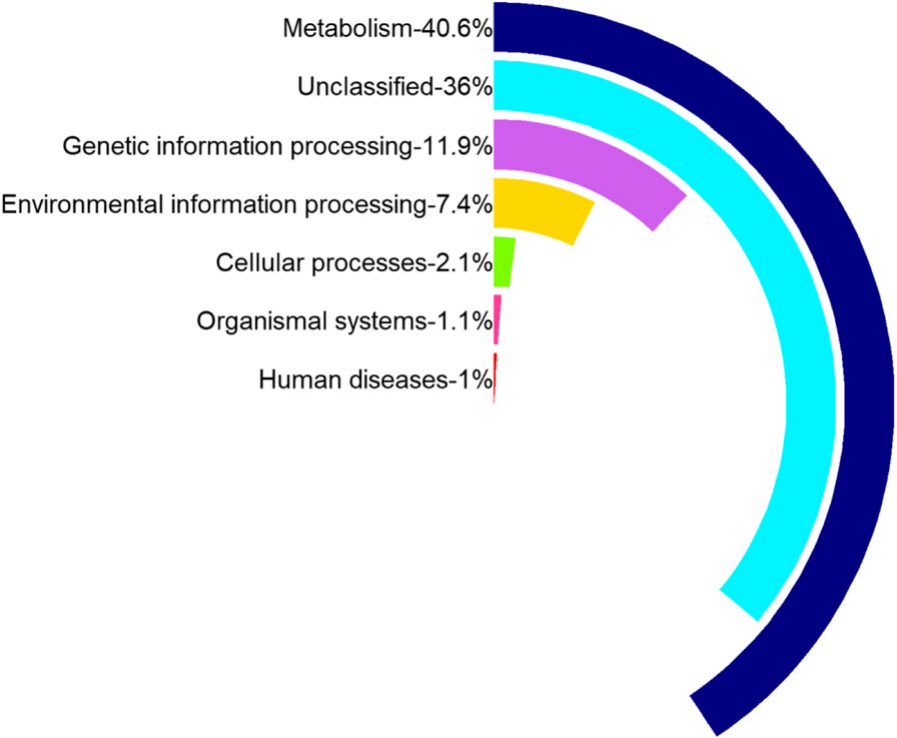
The relative number of genes assigned to KEGG functional modules

Metabolic potential analysis was performed by mapping reads to the Kyoto Encyclopedia of Genes and Genomes (KEGG) database based on six functional modules (metabolism, genetic information processing, environmental information processing, cellular processes, organismal systems, and human diseases). The results indicated that metabolism-related genes were the most represented in this community, accounting for 40.6% of the dataset (Fig. 3), followed by genes that are involved in genetic information processing (11.9%) (e.g. transcription, translation, replication and repair), environmental information processing (7.4%) (membrane transport, signal transduction, and signaling molecules and interaction), cellular processes (2.1%) (e.g. transport and catabolism, cell motility, cell growth and death), organismal systems (1.1%) (e.g. environmental adaptation, immune system, circulatory systems), and human diseases (1.0%) (e.g. infectious diseases, metabolic diseases, neurodegenerative diseases) (Fig. 3, Additional file 5: Table S1).

**Fig. 4:**
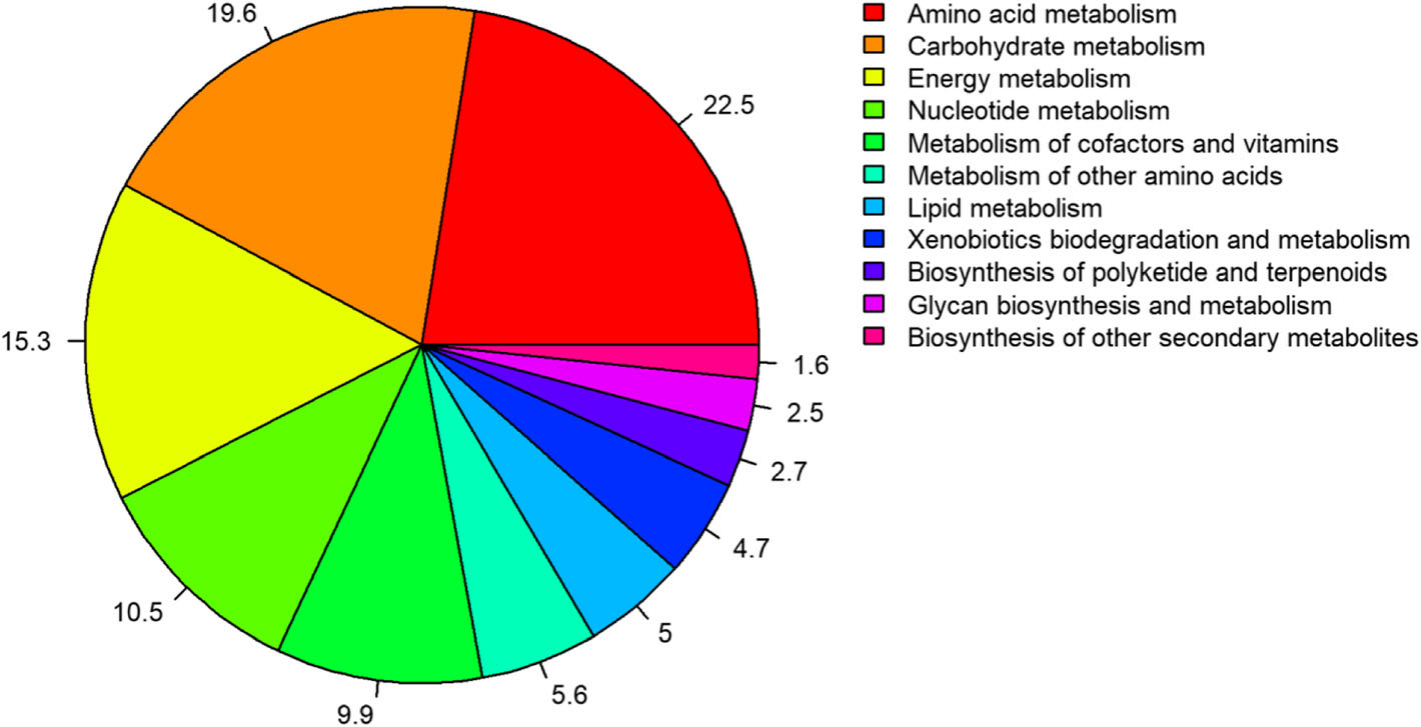
The relative number of genes assigned to metabolism

A deeper analysis of the metabolism function module revealed that the most abundant genes were for the metabolism of amino acids (22.5%) (e.g. alanine, aspartate, glutamate, glycine, serine and threonine), followed closely by carbohydrate metabolism (19.6%) (e.g. glycolysis/gluconeogenesis, citrate cycle (TCA cycle), pentose phosphate pathway, starch and sucrose metabolism), energy metabolism (15.3%) (e.g. oxidative phosphorylation, methane metabolism, nitrogen metabolism), nucleotide metabolism (10.5%) (purine and pyrimidine metabolism), metabolism of cofactors and vitamins (9.9%) (e.g. porphyrin and chlorophyll metabolism, pantothenate and CoA biosynthesis), metabolism of other amino acids (5.6%) (e.g. selenocompound, glutathione and cyanoamino acid), lipid metabolism (5.0%) (e.g. fatty acid degradation, fatty acid biosynthesis, glycerophospholipid metabolism), xenobiotics biodegradation and metabolism (4.7%) (e.g. benzoate, 3-chloroacrylic acid and nitrotoluene degradation), glycan biosynthesis and metabolism (2.5%) (e.g. peptidoglycan biosynthesis, lipopolysaccharide biosynthesis, glycosaminoglycan degradation), biosynthesis of polyketides and terpenoids (2.7%) (e.g. terpenoid backbone biosynthesis, limonene and pinene degradation, biosynthesis of ansamycins), and biosynthesis of other secondary metabolites (1.6%) (e.g. streptomycin, novobiocin, and isoquinoline alkaloid biosynthesis) (Fig. 4, Additional file 5: Table S1, Additional file 6: Table S2).

**Fig. 5:**
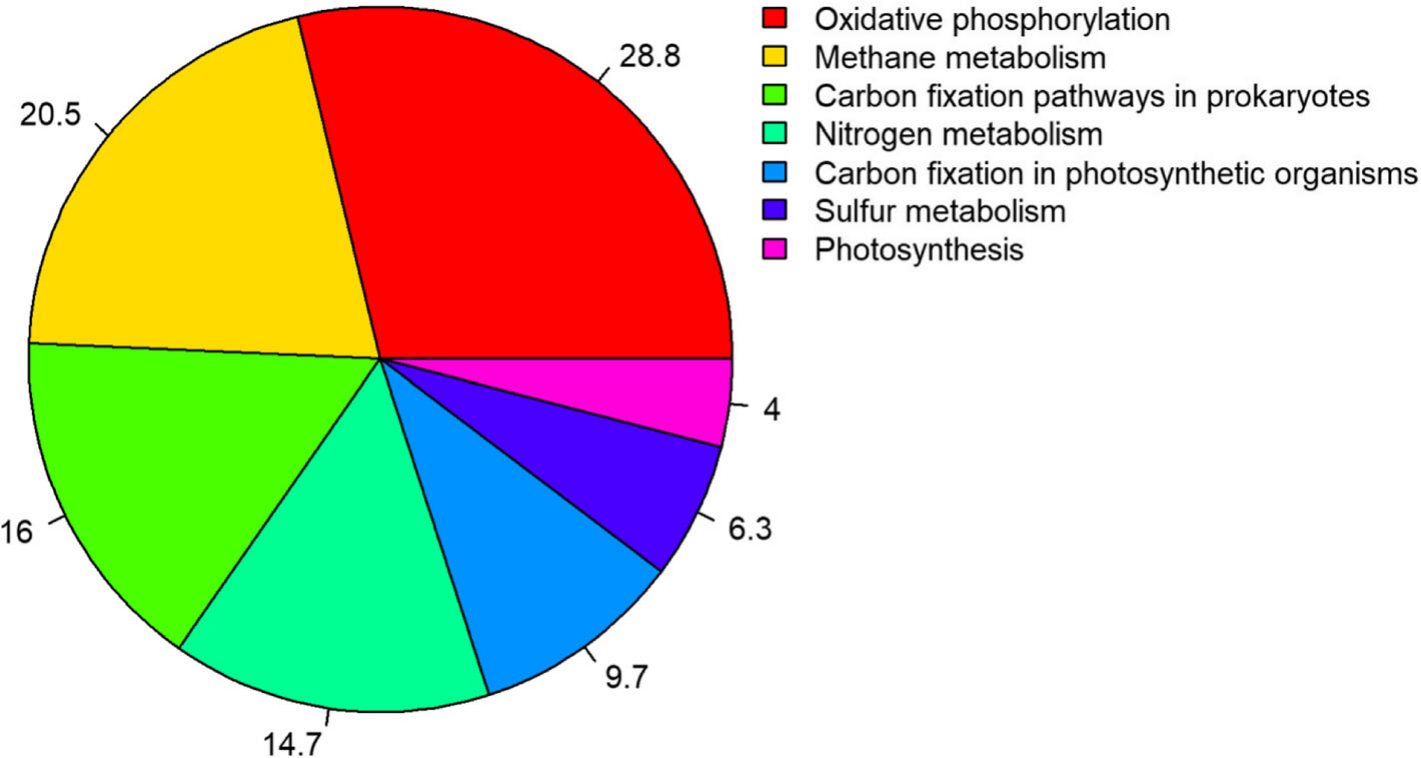
The relative number of genes assigned to energy metabolism

**Fig. 6:**
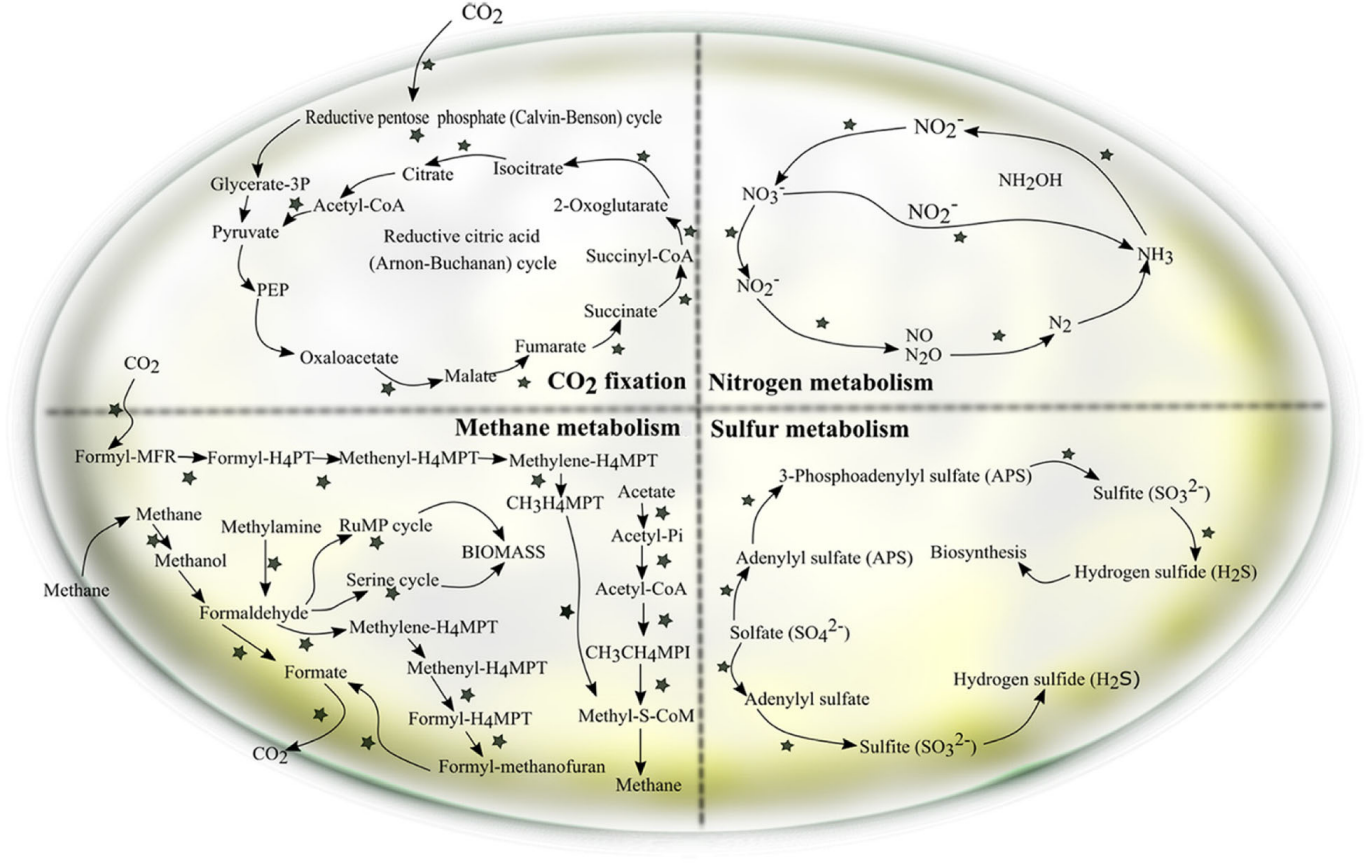
Overview of the identified energy metabolism of the Manao-Pee cave soil community based on the KEGG database. Stars indicate the presence of genes that are responsible for a particular pathway

As metabolism of amino acids and carbohydrates are common in all life forms, and to better understand how microbes thrive under energetically unfavorable conditions such as in caves, we therefore focused on other energy-producing metabolic pathways. Our results showed that the most abundant genes for these pathways are related to oxidative phosphorylation (28.8%) (e.g. NADH-quinone oxidoreductase, cytochrome c oxidase, succinate dehydrogenase/fumarate reductase, flavoprotein subunit) (Fig. 5, Additional file 6: Table S2, Additional file 7: Table S3), and methane metabolism (20.5%). Some of the identified functional genes that may encode by methanogens are: 5,10-methylenetetrahydromethanopterin reductase, tetrahydromethanopterin S-methyltransferase, and acetate kinase; and those encoded by methanotrophs are: methane monooxygenase and formate dehydrogenase (Fig. 5, Fig. 6, Additional file 6: Table S2, Additional file 8: Table S4). Also, genes involved in metabolism of carbon particularly of carbon fixation in prokatyotes were also detected (16.0%) (e.g. 2-oxoglutarate ferredoxin oxidoreductase, pyruvate ferredoxin oxidoreductase, isocitrate dehydrogenase) (Fig. 5, Fig. 6, Additional file 6: Table S2, Additional file 9: Table S5). In addition, microbial genes involved in carbon fixation in photosynthetic organisms (9.7%) (e.g. ribulose-bisphosphate carboxylase, fructose-bisphosphate aldolase, ribose 5-phosphate isomerase) (Fig. 5, Additional file 6: Table S2, Additional file 10: Table S6) and photosynthetic pathway (4.0%) (e.g. F-type H^+^-transporting ATPase, cytochrome b6-f complex iron-sulfur subunit, photosystem II oxygen-evolving enhancer protein 1) were also detected in this community (Fig. 5, Additional file 6: Table S2, Additional file 11: Table S7). Moreover, the results also revealed functional genes involved in the nitrogen cycle (14.7%) (Fig. 5, Fig. 6, Additional file 6: Table S2). The identified functional genes would be responsible for nitrification (e.g. ammonia monooxygenase, hydroxylamine dehydrogenase), denitrification (e.g. nitrate reductase, nitrite reductase (NO-forming), periplasmic nitrate reductase), assimilatory and dissimilatory nitrate reduction (e.g. assimilatory nitrate reductase, nitrite reductase (NADH), ferredoxin-nitrite reductase) (Additional file 12: Table S8). Last but not least, microbial genes involved in sulfur metabolism (6.3%) (e.g. sulfite reductase (NADPH) flavoprotein alpha and beta components, sulfite reductase (ferredoxin), sulfate adenylytransferase) were also found (Fig. 5, Fig. 6, Additional file 6: Table S2, Additional file 13: Table S9).

Apart from the primary metabolites, microbial genes involved in other biological pathways of secondary metabolites were also identified. Specifically, 4.7% of the genes under the metabolism function module were for xenobiotic biodegradation and metabolism (Fig. 4, Additional file 5: Table S1). Among them, benzoate degradation via CoA ligation was the most abundant (19.5%) (Additional file 6: Table S2). Other degradation pathways identified were: nitrotoluene degradation (7.3%), geraniol degradation (6.2%), 3-chloroacrylic acid degradation (5.9%), other benzoate degradation (5.1%), polycyclic aromatic hydrocarbon degradation (4.5%), styrene degradation (4.4%), chlorocyclohexane and chlorobenzene degradation (3.9%), 1,2-dichloroethane degradation (3.8%), caprolactam degradation (3.5%), atrazine degradation (2.9%), aminobenzoate degradation (2.3%), carbazole degradation (2.0%), xylene degradation (1.6%), ethylbenzene degradation (1.6%), fluorobenzoate degradation (1.6%), toluene degradation (1.5%), and fluorene degradation (1.0%). Moreover, metabolism of xenobiotics by cytochrome P450 (4.2%), drug metabolism - cytochrome P450 (4.5%), and drug metabolism - other enzymes (11.0%) were also detected (Additional file 6: Table S2). Except for xenobiotic biodegradation and metabolism, microbial genes involved in metabolism of terpenoids and polyketides were also detected (2.7%) (Fig. 4, Additional file 5: Table S1). The most abundant biological pathway in metabolism of terpenoids and polyketides was terpenoid backbone biosynthesis (43.8%) (Additional file 6: Table S2), followed by limonene and pinene degradation (14.0%), biosynthesis of ansamycins (11.5%), polyketide sugar unit biosynthesis (11.3%), tetracycline biosynthesis (7.1%), biosynthesis of vancomycin group antibiotics (6.0%), biosynthesis of siderophore group nonribosomal peptides (2.45%), zeatin biosynthesis (2.0%), and carotenoid biosynthesis (1.1%) (Additional file 6: Table S2). In addition, biosynthetic pathways of other compounds were also identified in this community (1.6%) (Fig. 4, Additional file 5: Table S1), namely, streptomycin biosynthesis (35.1%), novobiocin biosynthesis (14.1%), tropane, piperidine and pyridine alkaloid biosynthesis (13.5%), phenylpropanoid biosynthesis (13.1%), isoquinoline alkaloid biosynthesis (9.5%), penicillin and cephalosporin biosynthesis (5.1%), butirosin and neomycin biosynthesis (3.9%), stilbenoid, diarylheptanoid and gingerol biosynthesis (1.4%), flavonoid biosynthesis (1.2%), and betalain biosynthesis (1.1%) (Additional file 6: Table S2).

## Discussion

### Analysis of microbial diversity in the cave soil community

Cave habitats, in general, have unfavorable conditions to the development and support of life; nonetheless, caves do harbor a considerable diversity of microorganisms. Different groups of microorganisms can be found in caves such as bacteria, archaea, viruses, fungi, and protozoa [21, 23]. It has been suggested that “everything is everywhere, but the environment selects” [35, 36]. This would thus suggest that the unique characteristics of caves, i.e. their geological locations and physico-chemical parameters, may influence microbial community structures and their ecological activity.

Previously, bacterial diversity in Manao-Pee cave soil community was explored using amplicon sequencing of 16S rRNA gene (V5-V6 regions) [27]. The microbial community structure of soil sample inside and outside the cave were totally different [27]. This suggested that conditions within caves can exclude those microbes that are not adapted to thrive under specific cave conditions. Consistent with the 16S rRNA-based community structure study, the shotgun metagenomic sequencing performed in the current study confirmed that *Actinobacteria* and *Proteobacteria* were the dominant bacterium phyla in Manao-Pee cave community (Additional file 14: Figure S5). Our observation is consistent with other studies which suggest that these two phyla are the cosmopolitan groups of bacteria in cave ecosystems [23, 24, 37, 38]. However, at a deeper taxonomic level, shotgun metagenomic sequencing provided increased resolution, enabling the detection of more microbial taxonomic profiles than 16S rRNA sequencing [27], especially of rare microorganisms. For example, at family level, 123 bacterial families were identified by shotgun sequencing, but only 55 families were detected by amplicon sequencing (Additional file 15: Figure S6a-d).

#### Actinobacteria

*Actinobacteria* are found in a wide variety of habitats including extreme environments [39–41]. Their success in these environments suggested that they have broad adaptive abilities, such as the capability of producing special metabolites (e.g. metal chelators, and antimicrobial compounds), as well as their profligate of secreted hydrolytic enzymes that may help in obtaining nutrient sources from various substrates [42, 43]. Furthermore, this group of bacteria also play an ecologically significant role in several ecological processes such as biogeochemical cycles (e.g nitrogen fixation, sulfur oxidization), bioremediation (e.g. clean up of soil contaminated with polycyclic aromatic hydrocarbons), bioweathering (e.g. speleothems formation), and plant growth promotion (e.g production of plant growth regulators, production of siderophores to enhance iron availability, promotion of symbiosis between nitrogen fixing bacteria or mycorrhiza and plants) [23, 39, 44–49]. In cave microbiomes, they are not only found dominating in soil and sediments [24], but are also the prominent group on the cave walls, stalactites, and stalagmites [21, 38]. Using scanning electron microscopy, members of *Actinobacteria* are found to promote mineral dissolution and precipitation [23, 50], suggesting that this group of bacteria play a significant role in the formation of secondary mineral deposits which was previously viewed as an abiotic reaction. As the *Actinobacteria* phylum constitute the bulk of cave microbial communities, such habitats appear to favour this group of bacteria [23]. It has been speculated that their highly prolific source of secondary metabolites could be a driving factor for them to thrive under energetically unfavorable and nutrient-limited conditions [42].

*Actinobacteria* especially of the genus *Streptomyces* have long been recognized to produce pharmaceutically important bioactive molecules [51, 52]. As there is a decline in the discovery of novel bioactive molecules from *Streptomyces* in the past two decades [53], intensive efforts have focused on finding bioactive molecules derived from *Actinobacteria* from extreme or underexplored habitats such as caves, deserts, hot springs, and deep-sea sediments [39, 42, 54]. In this study, apart from *Streptomyces* spp*.*, we also identified the presence of rare *Actinobacteria* (e.g. *Mycobacterium*, *Nocardioides*, *Marmoricola*, *Crossiella*, *Amycolatopsis*, *Nocardia*, *Pseudonocardia*, and *Saccharopolyspora*) in Manao-Pee cave. These groups of microorganisms are of particular interest as they are likely to harbor biosynthetic pathways of novel bioactive compounds [27, 55].

#### Proteobacteria

It is known that *Proteobacteria* constitutes the largest and most diverse group within the domain bacteria [56]. This group inhabits diverse ecological niches [57], and is one of the most prevalent groups in cave ecosystems [21, 24, 58]. It is also known that *Proteobacteria* play an important ecological role in energy generating metabolisms (e.g. carbon fixation, nitrogen metabolism and sulfur metabolisms) [56, 57]. In the current study, among the five classes of *Proteobacteria*, the *Gammaproteobacteria* was the most prevalent in Mano-Pee cave soil community, with the most common genus belonging to *Salinisphaera* (6.6%)*, Lysobacter* (6.1%), and *Xanthomonas* (5.5%). It has been well documented that this class harbors metabolically and ecologically diverse chemolithoautotrophs that can use a variety of inorganic molecules as an electron source [21]. Thus, they might play a vital role in sustaining diverse groups of other microorganisms in this ecosystem.

### Metabolic potential analysis of Manao-Pee cave soil community

A metabolic potential analysis provides new opportunities to bridge the knowledge gap in the field of microbial ecology. As opposed to microbial profiling information, knowledge regarding metabolic potential of cave-dwelling microorganisms is very limited. The results of such a study will provide molecular biological evidence to increase our understanding of how cave microorganisms adapt and thrive under unfavorable conditions. Moreover, a metabolic potential investigation of cave microorganisms may also be applicable for applied research as well since the information obtained should provide leads to novel and useful microbial metabolites.

### Energy metabolism

Obtaining sufficient energy is one of most important ecological processes for survival of any organism. Ecologically, it is generally known that photosynthesis is the primary energy producing process on our planet [25]. Microbial communities in terrestrial ecosystems are also directly or indirectly dependent on energy and organic carbon that ultimately originated from this process. One of a few exceptions is the cave ecosystem. Devoid of light, although allochonous organic material can derive from photic surface environment [21, 42], microbes have to rely on alternate non-photosynthetic primary production strategies in order to thrive under unfavourable conditions. It has been reported that in caves, chemolithoautotrophic microbes are important as they obtain energy from the oxidation of inorganic molecules which in turn support the growth of other microbial populations [59]. Our study showed that the most abundant functional genes were for oxidative phosphorylation. This is not surprising, as it is the major metabolic pathway providing energy for the majority of aerobic organisms. Moreover, the microbial genes encoding methane metabolism, carbon fixation, nitrogen metabolism, sulfur metabolism, and photosynthesis were also identified in the cave soil community.

### Methane metabolism

Methane is one of the key elements in the global carbon cycle. Some microorganisms can obtain their energy from methane production, some can even use this molecule as an energy and carbon source [60, 61]. Our results suggesedt that methanogenic (e.g. *Euryarchaeota*, *Methylobacillus, Methylophaga*) and methanotrophic microbes (e.g. *Methylobacter*, *Methylococcus*, *Methylomicrobium*) can be found in the Manao-Pee cave soil community.

Methanogenic organisms can anaerobically obtain energy for growth by converting the limited number of substrates to methane as a metabolic byproduct. Most of these bacteria belong to the archaeal domain in the phylum *Euryarchaeota* [61] and can be found thriving in extreme habitats (e.g. deep soil sediments) [62]. It is known that there are three major methanogenesis pathways based on cabon sources (H_2_ + CO_2_ or formate (hydrogenotrophic methanogenesis), methyl-containing C-1 compounds (methylotrophic methanogenesis), and acetate (aceticlastic methanogenesis) [61, 63]. Due to the limited number of organic carbon sources in the caves, methane might be one of the energy-rich molecules that drive the microbial community.

Methanotrophic microorganisms can use methane as their sole carbon and energy source [60]. This biological pathway can be carried out under aerobic or anaerobic conditions. It has been reported that aerobic methanotrophic *Gammaproteobacteria*, *Alphaproteobacteria*, and *Verrucomicrobia* play a vital role in methane oxidation [64]. When oxygen is present, methane is converted to methanol and then to formaldehyde. Instead of being oxidized to carbon dioxide for energy generation, formaldehyde can also be assimilated into the cell’s biomass by means of ribulose monophosphate (RuMP) pathway or the serine pathway [60]. In this study, one of the key enzymes of this process, membrane-bound particulate methane monooxygenase (pMMO), which is the common MMO in aerobic methanotrophs was found. The results of our work suggested that methanogens and methanotrophs might play important ecological roles in sustaining diverse microbial communities in the Manao-Pee cave as well.

### Carbon fixation

Carbon fixation is an essential process in a microbial community through which inorganic carbon is incorporated into organic molecules [65]. In addition to photoautotrophic microorganisms, chemoautotrophic microorganisms can also carry out this process [65, 66]. So far, at least six autotrophic CO_2_ fixation pathways are known and different microorganisms generally utilize different fixation pathways [67]. All six CO_2_ fixation pathways are found on the speleothem surfaces in the Kartchner Caverns (Arizona, USA), with the predominant pathways being the Calvin-Benson-Bassham (CBB) and the reductive citric acid cycle (Arnon-Buchanan) [25]. In our study, genes encoding the reductive citric acid cycle were predominant (> 50%) in the Manao-Pee cave soil community. Surprisingly, since soil samples were collected from the dark areas deep inside the cave, functional genes involved in photosynthetic pathway and carbon fixation in photosynthetic organisms were also found. Concurrent with the result of microbial diversity analysis, algae and *Cyanobacteria* were also found in this community. They might contribute to the presence of the photosynthetic pathway as they usaually use this process to fix CO_2_ into biomass. [67]. Another possible reason is the presence of light-harvesting green-sulfur photoautotrophic bacteria (*Chlorbi*, *Chloroflexi*). It has been reported that these groups of microbes can obtain energy via photosynthesis at extremely low light intensities at which no other photosynthetic organisms can grow [68]. In addition, organisms capable of carrying out photosynthesis are also found in other phyla of bacteria: *Acidobacteria*, *Firmicutes*, *Gemmatimonadetes*, and *Proteobacteria* [69]. Members of those phyla that powered by light were also identified in this community (e.g. *Rhodospirillum*, *Ectothiorhodospira*, *Thiocapsa*). Moreover, it was reported that key proteins required for photosynthesis are also present in *Rubrobacter* (phylum *Actinobacteria*) [70]. Due to the physical characteristics of the Manao-Pee cave, those photoautotrophic microorganisms from the overlaying surface might enter the cave via the sinkhole. However, in the cave environment, photosynthetic genes may or may not be active, or the genes might exist and be only anciently presented within their genome.

### Nitrogen metabolism

Nitrogen is one of the most important elements for all life forms on our planet [71]. Microorganisms are the major contributor of nitrogen cycling, since it has been reported that atmospheric nitrogen, nitrate, nitrite, ammonium, and glutamine are widely used by microorganisms in environments [72]. However, most nitrogen on our planet is in the form of nitrogen gas (N_2_) which is not biologically available to support life [73] . Thus, nitrogen fixation by certain groups of bacteria and archaea (diazotrophs) is an important process by which atmospheric nitrogen is converted to ammonia available to other organisms [74]. From the study of the Frasassi caves (Italy) ecosystem, nitrogen fixation via nitrogenase activity is found in the cave waters [74]. However, in the present study, nitrogenase genes were not detected. Nonetheless, other subsystems related to nitrogen metabolism, including nitrification (e.g. ammonia monooxygenase, hydroxylamine dehydrogenase), denitrification (e.g. nitrate reductase, nitrite reductase (NO-forming), periplasmic nitrate reductase), assimilatory and dissimilatory nitrate reduction (e.g. assimilatory nitrate reductase, nitrite reductase (NADH), ferredoxin-nitrite reductase) were found in this community. These ecologically important processes might have critical roles in sustaining the microbial community within the cave ecosystem.

### Sulfur metabolism

Sulfur is another one the most abundant elements on Earth [75]. The biological transformation of inorganic sulfur can be performed by bacteria and archaea for the generation and conservation of biological energy [76]. These groups of microbes usually live in a symbiotic relationship with anaerobic methanotrophs as hydrogen generated from anaerobic methane oxidation can be used to drive redox reactions of inorganic sulfur [60]. However, in a soil habitat, sulfate (SO_4_^2−^) is the most oxidized and accessible form of sulfur for microoganisms [77]. In our study, the most identified genes (e.g. sulfite reductase, phosphoadenosine phosphosulfate reductase, sulfate adenylyltransferase, adenylylsulfate reductase) are responsible for sulfate reduction (energy consuming assimilatory pathway or energy producing dissimilatory pathway). Concurrent with microbial diversity information, sulfate-reducing microorganisms were also identified (e.g. *Desulfovibrio*, *Desulfonatronospira*, *Desulfatibacilium*, *Thermodesulfobium*). One of the identified genes in the assimilatory sulfate reduction pathway was sulfite reductase which catalyses the six-electron reduction of sulfite to hydrogen sulfide and water. This pathway is widely used by bacteria, fungi, and photosynthetic organisms to convert inorganic sulfate to sulfide which can be further incorporated into a carbon skeleton of sulfur-containing amino acids and proteins [78]. Apart from an energy consuming assimilatory pathway, microorganisms can also use sulfate as an alternative electron acceptor in the absence of oxygen. This biological pathway, dissimilatory sulfate reduction, serves as energy-yielding reactions for growth [75]. This suggested that in Manao-Pee cave, microorganisms involved in the sulfur cycle may also serve as one of the primary producers in the cave food web.

### Xenobiotic biodegradation and metabolism in Manao-Pee cave microbiome

Xenobiotic is a term referring to unnatural or foreign compounds, including polycyclic hydrocarbons, pollutants, antibiotics, polyaromatic, chlorinated and nitroaromatic compounds found in the biosphere [79, 80]. Some of these compounds can be very toxic, but can be transformed into less toxic compounds by various biological pathways [81, 82]. It has been reported that stress or low nutrient habitats can force living microoganisms to use any available nutrients to survive [83]; thus, cave-dwelling microroganisms may use the by-products of xenobiotic biodegradation for energy, nutrients, or final electron acceptors for their growth [79]. For instance, it has been reported that various bacteria (*Serratia* sp. KC1-MRL, *Bacillus liceniformis* KC2-MRL, *Bacillus* sp. KC3-MRL, *Stenotrophomonas* sp. KC4-MRL) isolated from soil samples of Kashmir Smast, a limestone cave in Khyber Pakhtoonkhuwa province, Pakistan, are capable of degrading polyethylene [83]. Therefore, it is also likely that xenobiotic biodegradation and metabolism might be another important process in sustaining diverse groups of microorganisms in Manao-Pee cave.

### Natural secondary metabolites production in Manao-Pee cave microbiome

In their natural environments, microbes usually form complex ecological networks, consisting of many species either directly or indirectly interacting with one another [84]. These networks interplay, potentially mediated by bacterial secondary metabolites, may result in successful establishment and maintenance of microbial populations [85].

Microorganisms produce a large variety of secondary metabolites. Even though production of these compounds is not considered essential for normal growth and development, it nonetheless provides essential ecological benefits [86]. Ecologically, secondary metabolites may contribute to both cooperative and competitive interactions among microoganisms [87]. Certain microbes, for example, produce secondary metabolites to compete with one another for limited resources [85]. Due to the nutrient-limited nature of a cave environment, it has been hypothesized that competition for natural resources might be the dominant adaptation of cave microbiome [37, 88]. Yet, at sub-optimal concentrations, secondary metabolites can also be used as signaling molecules (e.g. kanamycin, gentamycin, tetracycline) to help in inter- or intraspecies communications [85, 89]. In a cave microbiome, it is unlikely that all reactions necessary for growth and obtaining energy from complex inorganic molecules are carried out by a single species [21]. Thus, signaling molecules are needed to mediate cooperative and mutualistic actions among cave microorganisms; thereby, increasing the likelihood of survival under energetically unfavorable environments.

Among microbial secondary metabolites, antibiotics are of special interest. Due to the rise of antibiotic resistant pathogens [90], novel antibiotics discovery is essential. In recent years, there have been rigorous efforts to find new bioactive compounds from cave microbiomes because it is believed that the less studied cave environment might be a potential source for drug discovery [91–93].

## Conclusion

Microorganisms are key players in every environmental niches. Their taxonomic and functional distribution are selected for directly or indirectly by environmental factors. In this work we confirmed that Manao-Pee cave harbored a great diversity of bacteria, with the most dominant groups being *Actinobacteria* and *Gammaproteobacteria*. Metabolic functional analysis revealed microbial genes involved in various metabolic pathways. To survive under energetically unfavorable and nutrient-limited conditions, methane metabolism, carbon fixation, nitrogen metabolism, sulfur metabolism, xenobiotic biodegradation and metabolism, and secondary metabolite production all might play important ecological roles in sustaining the diverse groups of microorganisms. Beyond providing information on microbial diversity and associated metabolic potential for survival under the cave conditions and to better understand life in the hidden world, our study also suggested that unique bioactive molecules with promising activity in medical and industrial processes may also be obtained from Manao-Pee cave.

## Additional files

Additional file 1: **Figure S1.** Distribution of *Actinobacteria* in the soil community of Manao-Pee cave at (a) family level and (b) genus level. Percentage values represent the relative abundance of ribosomal RNA genes assigned to a particular taxon. (DOCX 623 kb)

Additional file 2: **Figure S2.** Distribution of *Proteobacteria* in the soil community of Manao-Pee cave at (a) class level, (b) family level, and (c) genus level. Percentage values represent the relative abundance of ribosomal RNA genes assigned to a particular taxon. (DOCX 761 kb)

Additional file 3: **Figure S3.** Distribution of archaeal phylum in the soil community of Manao-Pee cave. Percentage values represent the relative abundance of ribosomal RNA genes assigned to a particular taxon. (DOCX 110 kb)

Additional file 4: **Figure S4.** Distribution of eukaryotic organisms in the soil community of Manao-Pee cave. Percentage values represent the relative abundance of ribosomal RNA genes assigned to a particular taxon. (DOCX 147 kb)

Additional file 5: **Table S1.** The number of genes assigned to the various sub-functional modules. (DOCX 14 kb)

Additional file 6: **Table S2.** The relative number of genes assigned to the various biological pathways. (DOCX 27 kb)

Additional file 7: **Table S3.** The identified microbial genes involved in the oxidative phosphorylation pathway. (DOCX 17 kb)

Additional file 8: **Table S4.** The identified microbial genes involved in methane metabolism pathway. (DOCX 16 kb)

Additional file 9: **Table S5.** The identified microbial genes involved in carbon fixation pathways in prokaryotes. (DOCX 15 kb)

Additional file 10: **Table S6.** The identified microbial genes involved in carbon fixation from photosynthetic organisms. (DOCX 14 kb)

Additional file 11: **Table S7.** The identified microbial genes involved in the photosynthetic pathway. (DOCX 14 kb)

Additional file 12: **Table S8.** The identified microbial genes involved in nitrogen metabolism pathway. (DOCX 16 kb)

Additional file 13: **Table S9.** The identified microbial genes involved in sulfur metabolism pathway. (DOCX 15 kb)

Additional file 14: **Figure S5.** Comparison of bacterial diversity in the soil community of Manao-Pee cave at the phylum level by 16S rRNA sequencing versus shotgun metagenomic sequencing. Both techniques, at this taxonomic depth, gave comparable results, revealing that most Manao-Pee cave microbes belonged to the phyla *Actinobacteria* or *Proteobacteria*. (DOCX 950 kb)

Additional file 15: **Figure S6.** Comparison of bacterial diversity in the soil community of Manao-Pee cave at (a) class, (b) order, (c) family, and (d) genus level by amplicon sequencing versus shotgun metagenomic sequencing. In general, at deeper taxonomic levels, shotgun metagenomic sequencing gave more insight into the numbers and identity of microbial present. (DOCX 12712 kb)

## Data Availability

All datasets present in the research article were submitted to the NCBI Sequence Read Archive under the accession number PRJNA485054.

## Abbreviations

eDNA: Environmental DNA
KEGG: Kyoto Encyclopedia of Genes and Genomes
OTU: Operational taxonomic unit
rRNA: Ribosomal RNA
