# Supplementary material for: Shotgun metagenomic sequencing from Manao-Pee cave, Thailand, reveals insight into the microbial community structure and its metabolic potential
Source: BMC Microbiol. 2019 Jun 27;19:144. doi: 10.1186/s12866-019-1521-8 (PMC6598295; doi:10.1186/s12866-019-1521-8)
Supplement: Supplementary file 1 — Figure S1. Distribution of Actinobacteria in the soil community of Manao-Pee cave at (a) family level and (b) genus level. Percentage values represent the relative abundance of ribosomal RNA genes assigned to a particular taxon. (DOCX 623 kb) [file 12866_2019_1521_MOESM1_ESM.docx]

**Additional file 1: Figure S1.** Distribution of *Actinobacteria* in the soil community of Manao-Pee cave at (a) family level and (b) genus level. Percentage values represent the relative abundance of ribosomal RNA genes assigned to a particular taxon.


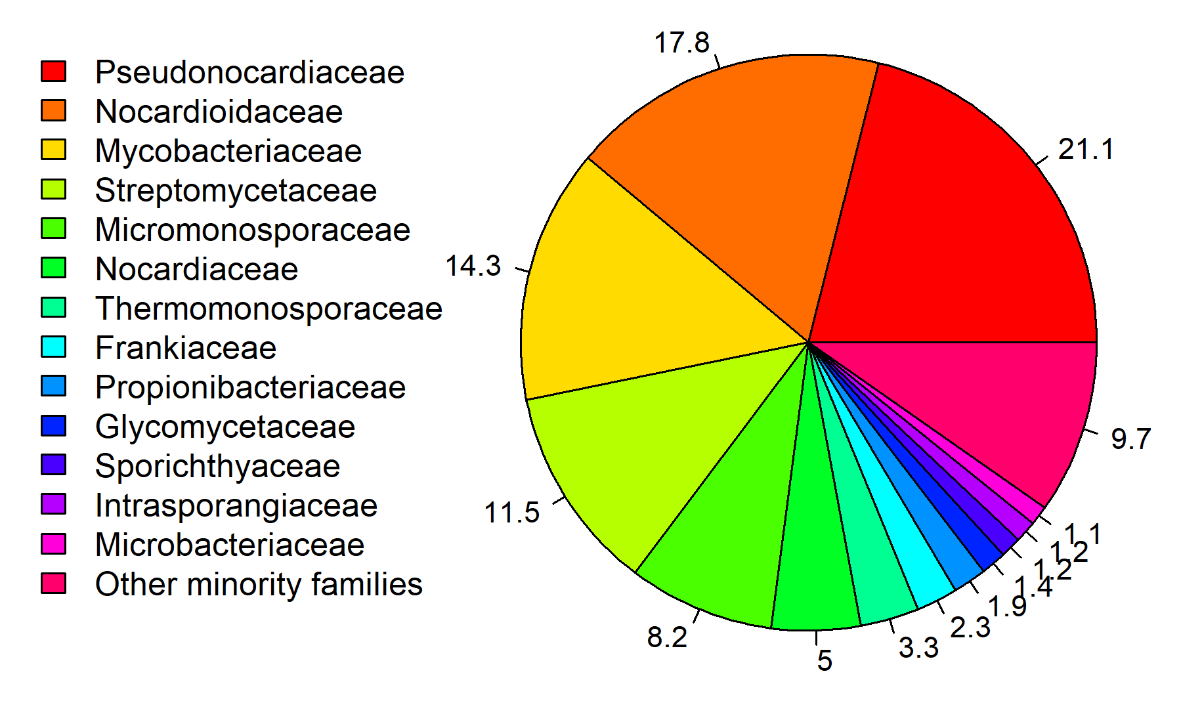


**a**


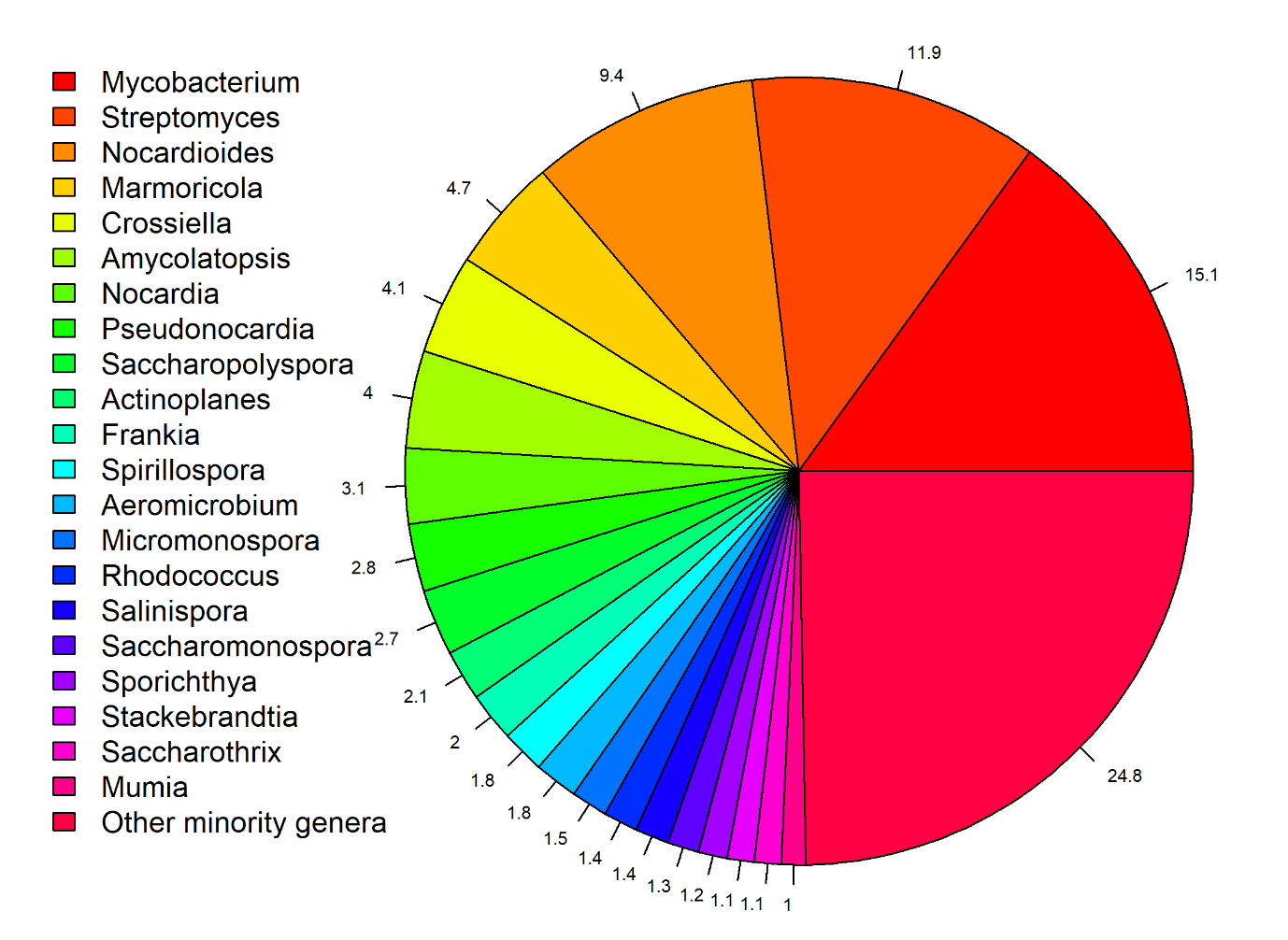


**b**
