# Supplementary material for: Shotgun metagenomic sequencing from Manao-Pee cave, Thailand, reveals insight into the microbial community structure and its metabolic potential
Source: BMC Microbiol. 2019 Jun 27;19:144. doi: 10.1186/s12866-019-1521-8 (PMC6598295; doi:10.1186/s12866-019-1521-8)
Supplement: Supplementary file 2 — Figure S2. Distribution of Proteobacteria in the soil community of Manao-Pee cave at (a) class level, (b) family level, and (c) genus level. Percentage values represent the relative abundance of ribosomal RNA genes assigned to a particular taxon. (DOCX 761 kb) [file 12866_2019_1521_MOESM2_ESM.docx]

**Additional file 2: Figure S2**. Distribution of *Proteobacteria* in the soil community of Manao-Pee cave at (a) class level, (b) family level, and (c) genus level. Percentage values represent the relative abundance of ribosomal RNA genes assigned to a particular taxon**.**


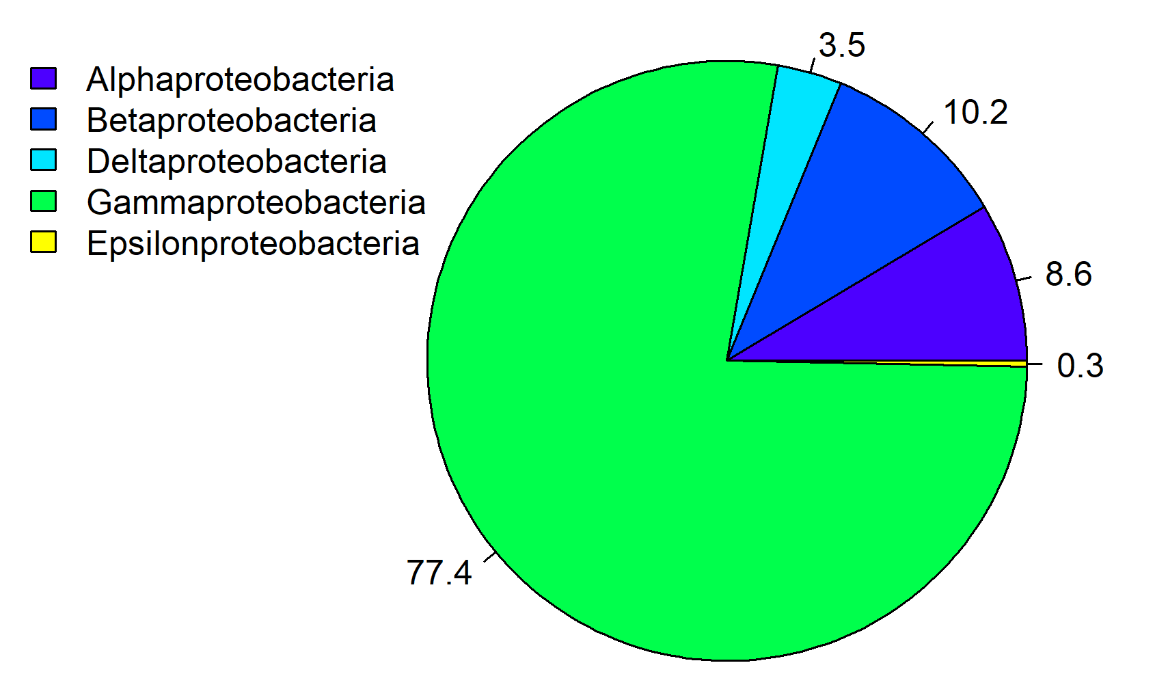


**a**

**b**


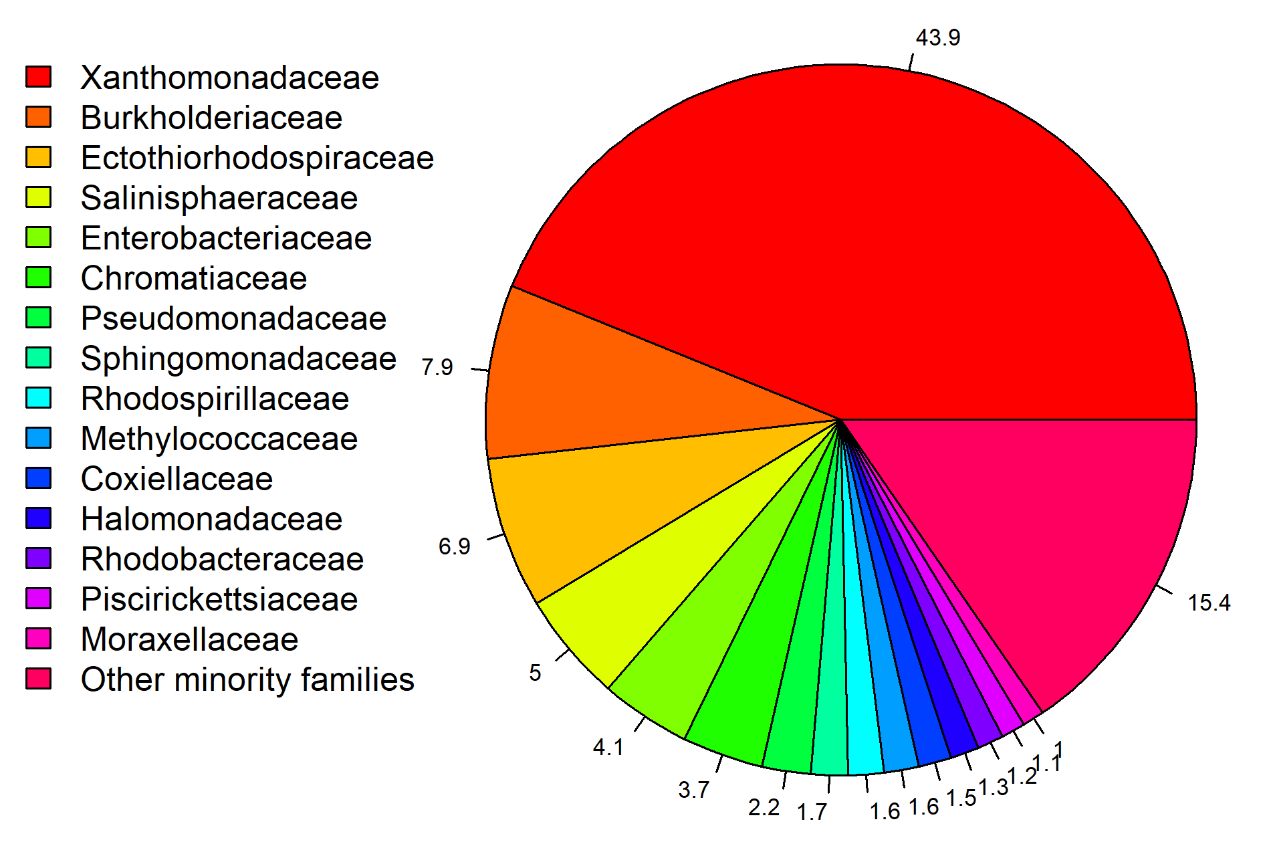


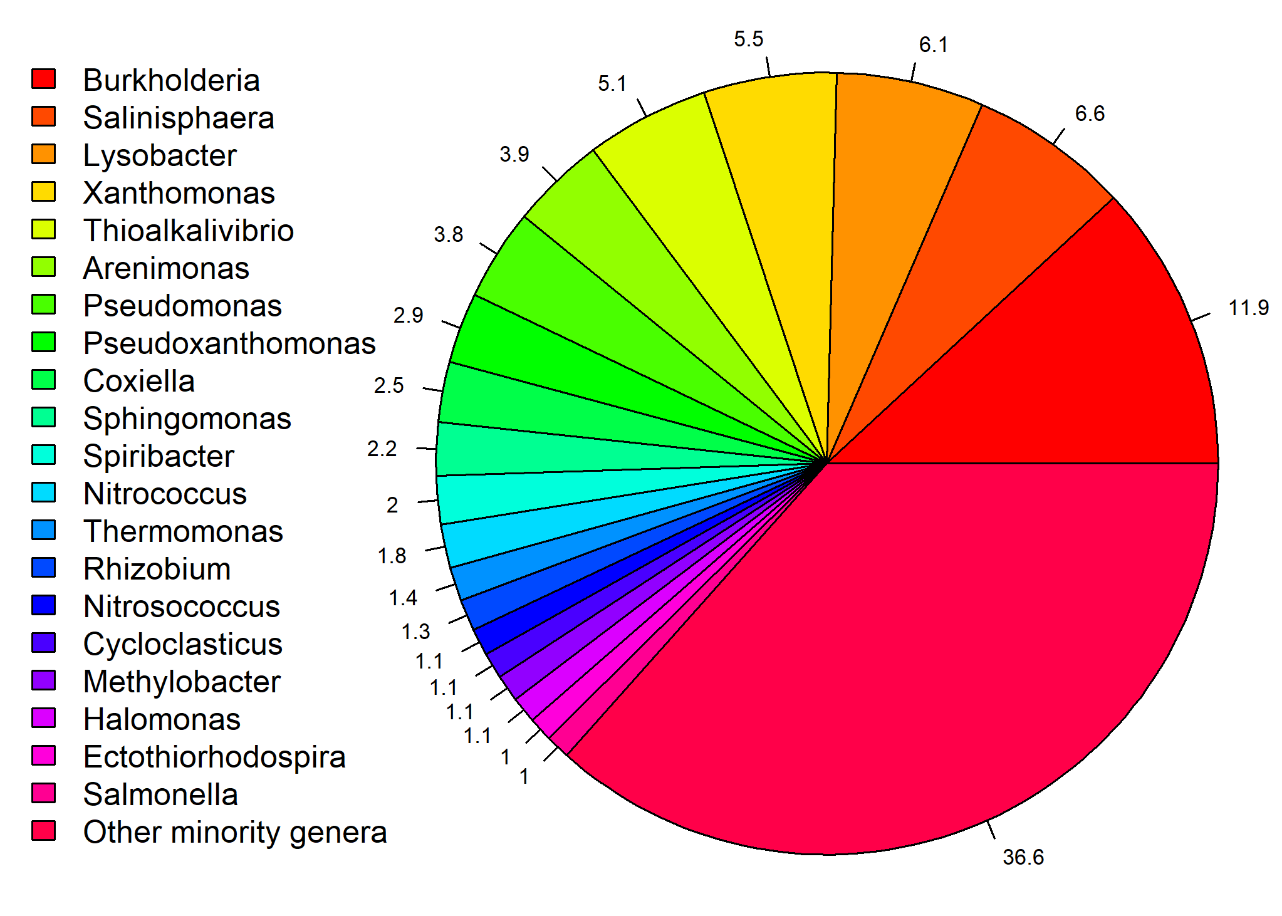


**c**
