# Supplementary material for: Shotgun metagenomic sequencing from Manao-Pee cave, Thailand, reveals insight into the microbial community structure and its metabolic potential
Source: BMC Microbiol. 2019 Jun 27;19:144. doi: 10.1186/s12866-019-1521-8 (PMC6598295; doi:10.1186/s12866-019-1521-8)
Supplement: Supplementary file 3 — Figure S3. Distribution of archaeal phylum in the soil community of Manao-Pee cave. Percentage values represent the relative abundance of ribosomal RNA genes assigned to a particular taxon. (DOCX 110 kb) [file 12866_2019_1521_MOESM3_ESM.docx]

**
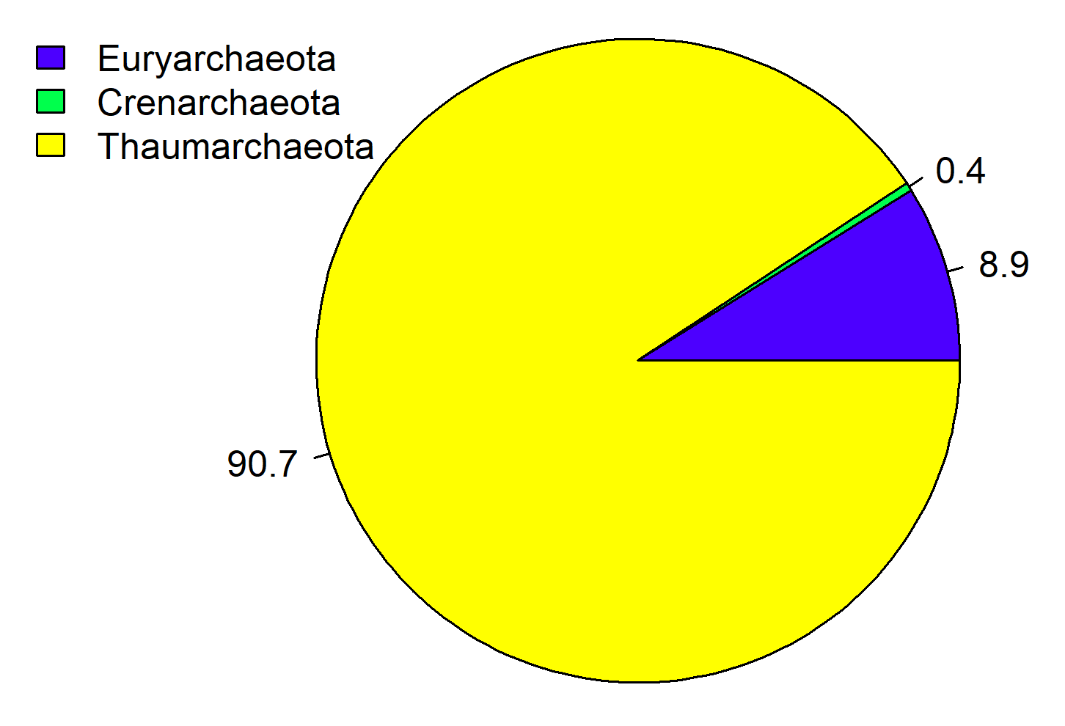
Additional file 3: Figure S3.** Distribution of archaeal phylum in the soil community of Manao-Pee cave. Percentage values represent the relative abundance of ribosomal RNA genes assigned to a particular taxon**.**
