# Supplementary material for: Shotgun metagenomic sequencing from Manao-Pee cave, Thailand, reveals insight into the microbial community structure and its metabolic potential
Source: BMC Microbiol. 2019 Jun 27;19:144. doi: 10.1186/s12866-019-1521-8 (PMC6598295; doi:10.1186/s12866-019-1521-8)

**Additional file 4: Figure S4**. Distribution of eukaryotic organisms in the soil community of Manao-Pee cave. Percentage values represent the relative abundance of ribosomal RNA genes assigned to a particular taxon**.**


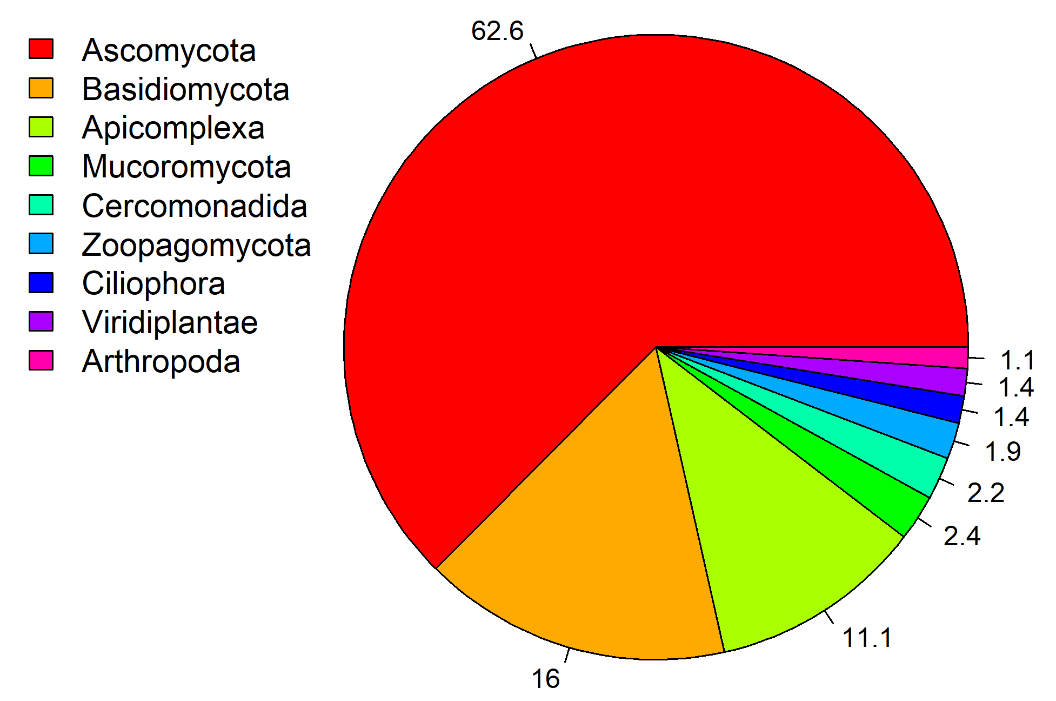

Supplement: Supplementary file 4 — Figure S4. Distribution of eukaryotic organisms in the soil community of Manao-Pee cave. Percentage values represent the relative abundance of ribosomal RNA genes assigned to a particular taxon. (DOCX 147 kb) [file 12866_2019_1521_MOESM4_ESM.docx]
