# Supplementary material for: Shotgun metagenomic sequencing from Manao-Pee cave, Thailand, reveals insight into the microbial community structure and its metabolic potential
Source: BMC Microbiol. 2019 Jun 27;19:144. doi: 10.1186/s12866-019-1521-8 (PMC6598295; doi:10.1186/s12866-019-1521-8)
Supplement: Supplementary file 5 — Table S1. The number of genes assigned to the various sub-functional modules. (DOCX 14 kb) [file 12866_2019_1521_MOESM5_ESM.docx]

**Additional file 5: Table S1**. The number of genes assigned to the various sub-functional modules.

| **Functional module** | **Pathway** | **MPI** |
| --- | --- | --- |
| Metabolism | Carbohydrate metabolism | 96342 (19.6%) |
|  | Energy metabolism | 75577 (15.3%) |
|  | Lipid metabolism | 24790 (5.0%) |
|  | Nucleotide metabolism | 51632 (10.5%) |
|  | Amino acid metabolism | 111032 (22.5%) |
|  | Metabolism of other amino acids | 27555 (5.6%) |
|  | Glycan biosynthesis and metabolism | 12494 (2.5%) |
|  | Metabolism of cofactors and vitamins | 48868 (9.9%) |
|  | Biosynthesis of polyketides and terpenoids | 13314 (2.7%) |
|  | Biosynthesis of other secondary metabolites | 7903 (1.6%) |
|  | Xenobiotics biodegradation and metabolism | 23214 (4.7%) |
| Genetic information processing | Transcription | 9687 (10.0%) |
|  | Translation | 42976 (42.2%) |
|  | Folding, sorting and degradation | 18994 (19.5%) |
|  | Replication and repair | 25606 (26.3%) |
| Environmental information processing | Membrane transport | 43061 (70.3%) |
|  | Signal transduction | 18166 (29.7%) |
|  | Signaling molecules and interaction | 2 (0.0%) |
| Cellular processes | Transport and catabolism | 5801 (34.3%) |
|  | Cell motility | 4212 (24.9%) |
|  | Cell growth and death | 6874 (40.7%) |
|  | Cell communication | 14 (0.1%) |
| Organismal systems | Immune system | 596 (5.2%) |
|  | Endocrine system | 5409 (47.1%) |
|  | Circulatory system | 381 (3.3%) |
|  | Excretory system | 1677 (14.6%) |
|  | Nervous system | 13 (0.1%) |
|  | Sensory system | 4 (0.0%) |
|  | Development | 7 (0.1%) |
|  | Environmental adaptation | 3393 (29.6%) |
| Human diseases | Cancers | 1206 (14.8% |
|  | Immune system diseases | 339 (4.1%) |
|  | Neurodegenerative diseases | 2465 (30.2%) |
|  | Cardiovascular diseases | 232 (2.8%) |
|  | Metabolic diseases | 2282 (27.9%) |
|  | Infectious diseases | 1647 (20.2%) |
