# Supplementary material for: Shotgun metagenomic sequencing from Manao-Pee cave, Thailand, reveals insight into the microbial community structure and its metabolic potential
Source: BMC Microbiol. 2019 Jun 27;19:144. doi: 10.1186/s12866-019-1521-8 (PMC6598295; doi:10.1186/s12866-019-1521-8)
Supplement: Supplementary file 6 — Table S2. The relative number of genes assigned to the various biological pathways. (DOCX 27 kb) [file 12866_2019_1521_MOESM6_ESM.docx]

**Additional file 6: Table S2.** The relative number of genes assigned to the various biological pathways.

| **Biological pathway** | **Pathway** | **MPI** |
| --- | --- | --- |
| Carbohydrate metabolism | Glycolysis/gluconeogenesis | 17817 (11.2%) |
|  | Citrate cycle (TCA cycle) | 19903 (12.6%) |
|  | Pentose phosphate pathway | 9704 (6.1%) |
|  | Pentose and glucuronate interconversions | 3640 (2.3%) |
|  | Fructose and mannose metabolism | 5803 (3.7%) |
|  | Galactose metabolism | 5849 (3.7%) |
|  | Ascorbate and aldarate metabolism | 2053 (1.3%) |
|  | Starch and sucrose metabolism | 9841 (6.2%) |
|  | Amino sugar and nucleotide sugar metabolism | 13742 (8.7%) |
|  | Pyruvate metabolism | 21939 (13.8%) |
|  | Glyoxylate and dicarboxylate metabolism | 9841 (6.2%) |
|  | Propanoate metabolism | 13492 (8.5%) |
|  | Butanoate metabolism | 15771 (10.0%) |
|  | C5-Branched dibasic acid metabolism | 5411 (3.4%) |
|  | Inositol phosphate metabolism | 3669 (2.3%) |
| Energy metabolism | Oxidative phosphorylation | 25960 (28.8%) |
|  | Photosynthesis | 3643 (4.0%) |
|  | Photosynthesis - antenna proteins | 8 (0.0%) |
|  | Carbon fixation in photosynthetic organisms | 8756 (9.7%) |
|  | Carbon fixation pathways in prokaryotes | 14398 (16.0%) |
|  | Methane metabolism | 18447 (20.5%) |
|  | Nitrogen metabolism | 13264 (14.7%) |
|  | Sulfur metabolism | 5654 (6.3%) |
| Lipid metabolism | Fatty acid biosynthesis | 4903 (15.2%) |
|  | Fatty acid elongation | 15 (0.0%) |
|  | Fatty acid degradation | 8501 (26.4%) |
|  | Synthesis and degradation of ketone bodies | 2583 (8.0%) |
|  | Steroid biosynthesis | 1217 (3.8%) |
|  | Primary bile acid biosynthesis | 137 (0.4%) |
|  | Secondary bile acid biosynthesis | 36 (0.1%) |
|  | Steroid hormone biosynthesis | 451 (1.4%) |
|  | Glycerolipid metabolism | 3912 (12.1%) |
|  | Glycerophospholipid metabolism | 4563 (14.2%) |
|  | Ether lipid metabolism | 719 (2.2%) |
|  | Sphingolipid metabolism | 1506 (4.7%) |
|  | Arachidonic acid metabolism | 951 (3.0%) |
|  | Linoleic acid metabolism | 140 (0.4%) |
|  | alpha-Linolenic acid metabolism | 389 (1.2%) |
|  | Biosynthesis of unsaturated fatty acids | 2201 (6.8%) |
| Nucleotide metabolism | Purine metabolism | 40613 (56.2%) |
|  | Pyrimidine metabolism | 31663 (43.8%) |
| Amino acid metabolism | Alanine, aspartate and glutamate metabolism | 20990 (14.2%) |
|  | Glycine, serine and threonine metabolism | 16570 (11.2%) |
|  | Cysteine and methionine metabolism | 14747 (10.0%) |
|  | Valine, leucine and isoleucine degradation | 12066 (8.2%) |
|  | Valine, leucine and isoleucine biosynthesis | 19129 (12.9%) |
|  | Lysine biosynthesis | 8115 (5.5%) |
|  | Lysine degradation | 5502 (3.7%) |

**Additional file 6: Table S2.** The relative number of genes assigned to each biological pathway (cont.).

| **Biological pathway** | **Pathway** | **MPI** |
| --- | --- | --- |
| Amino acid metabolism | Arginine and proline metabolism | 18689 (12.6%) |
|  | Histidine metabolism | 6410 (4.3%) |
|  | Tyrosine metabolism | 5028 (3.4%) |
|  | Phenylalanine metabolism | 4260 (2.9%) |
|  | Tryptophan metabolism | 7890 (5.3%) |
|  | Phenylalanine, tyrosine and tryptophan biosynthesis | 8630 (5.8%) |
| Metabolism of other amino acids | beta-Alanine metabolism | 4397 (14.4%) |
|  | Taurine and hypotaurine metabolism | 1850 (6.1%) |
|  | Phosphonate and phosphinate metabolism | 226 (0.7%) |
|  | Selenocompound metabolism | 10963 (35.9%) |
|  | Cyanoamino acid metabolism | 3642 (11.9%) |
|  | D-Glutamine and D-glutamate metabolism | 2039 (6.7%) |
|  | D-Arginine and D-ornithine metabolism | 85 (0.3%) |
|  | D-Alanine metabolism | 967 (3.2%) |
|  | Glutathione metabolism | 6378 (20.9%) |
| Glycan biosynthesis and metabolism | N-Glycan biosynthesis | 1118 (8.2%) |
|  | Various types of N-glycan biosynthesis | 1 (0.0%) |
|  | Other types of O-glycan biosynthesis | 2 (0.0%) |
|  | Glycosaminoglycan biosynthesis - chondroitin sulfate / dermatan sulfate | 0 (0.0%) |
|  | Glycosaminoglycan degradation | 710 (5.2%) |
|  | Glycosylphosphatidylinositol (GPI)-anchor biosynthesis | 9 (0.1%) |
|  | Glycosphingolipid biosynthesis - globo series | 925 (6.8%) |
|  | Glycosphingolipid biosynthesis - ganglio series | 367 (2.7%) |
|  | Lipopolysaccharide biosynthesis | 1874 (13.8%) |
|  | Peptidoglycan biosynthesis | 6825 (50.2%) |
|  | Other glycan degradation | 1765 (13.0%) |
| Metabolism of cofactors and vitamins | Thiamine metabolism | 3847 (7.8%) |
|  | Riboflavin metabolism | 1588 (3.2%) |
|  | Vitamin B6 metabolism | 2784 (5.7%) |
|  | Nicotinate and nicotinamide metabolism | 5452 (11.1%) |
|  | Pantothenate and CoA biosynthesis | 8064 (16.4%) |
|  | Biotin metabolism | 1486 (3.0%) |
|  | Lipoic acid metabolism | 786 (1.6%) |
|  | Folate biosynthesis | 2165 (4.4%) |
|  | One carbon pool by folate | 7055 (14.4%) |
|  | Retinol metabolism | 997 (2.0%) |
|  | Porphyrin and chlorophyll metabolism | 10924 (22.3%) |
|  | Ubiquinone and other terpenoid-quinone biosynthesis | 3886 (7.9%) |
| Metabolism of terpenoids and polyketides | Biosynthesis of ansamycins | 1633 (11.5%) |
|  | Biosynthesis of type II polyketide backbone | 30 (0.2%) |
|  | Tetracycline biosynthesis | 1013 (7.2%) |
|  | Polyketide sugar unit biosynthesis | 1605 (11.3%) |
|  | Biosynthesis of siderophore group nonribosomal peptides | 349 (2.5%) |
|  | Biosynthesis of vancomycin group antibiotics | 846 (6.0%) |
|  | Terpenoid backbone biosynthesis | 6208 (43.8%) |
|  | Monoterpenoid biosynthesis | 1 (0.0%) |

**Additional file 6: Table S2.** The relative number of genes assigned to each biological pathway (cont.).

| **Biological pathway** | **Pathway** | **MPI** |
| --- | --- | --- |
| Metabolism of terpenoids and polyketides | Limonene and pinene degradation | 1980 (14.0%) |
|  | Zeatin biosynthesis | 279 (2.0%) |
|  | Sesquiterpenoid and triterpenoid biosynthesis | 68 (0.5%) |
|  | Carotenoid biosynthesis | 149 (1.1%) |
| Biosynthesis of other secondary metabolites | Phenylpropanoid biosynthesis | 1393 (13.1%) |
|  | Stilbenoid, diarylheptanoid and gingerol biosynthesis | 150 (1.4%) |
|  | Flavonoid biosynthesis | 130 (1.2%) |
|  | Flavone and flavonol biosynthesis | 22 (0.2%) |
|  | Isoflavonoid biosynthesis | 1 (0.0%) |
|  | Indole alkaloid biosynthesis | 65 (0.6%) |
|  | Isoquinoline alkaloid biosynthesis | 1009 (9.5%) |
|  | Tropane, piperidine and pyridine alkaloid biosynthesis | 1426 (13.5%) |
|  | Caffeine metabolism | 84 (0.8%) |
|  | Betalain biosynthesis | 115 (1.1%) |
|  | Penicillin and cephalosporin biosynthesis | 540 (5.1%) |
|  | beta-Lactam resistance | 30 0.3% |
|  | Streptomycin biosynthesis | 3723 (35.1%) |
|  | Butirosin and neomycin biosynthesis | 418 (3.9%) |
|  | Clavulanic acid biosynthesis | 0 (0.0%) |
|  | Novobiocin biosynthesis | 1493 (14.1%) |
| Xenobiotics biodegradation and metabolism | Caprolactam degradation | 1238 (3.5%) |
|  | Dioxin degradation | 219 (0.6%) |
|  | Xylene degradation | 575 (1.6%) |
|  | Chlorocyclohexane and chlorobenzene degradation | 1368 (3.9%) |
|  | 3-Chloroacrylic acid degradation | 2079 (5.9%) |
|  | Trichloro-2,2-bis(4-chlorophenyl)ethane (DDT) degradation | 2 (0.0%) |
|  | Toluene degradation | 520 (1.5%) |
|  | 1,2-Dichloroethane degradation | 1340 (3.8%) |
|  | Chloroalkane and chloroalkene degradation | 42 (0.1%) |
|  | Styrene degradation | 1572 (4.4%) |
|  | Aminobenzoate degradation | 817 (2.3) |
|  | Naphthalene degradation | 341 (1.0%) |
|  | Ethylbenzene degradation | 557 (1.6%) |
|  | Fluorene degradation | 354 (1.0%) |
|  | Carbazole degradation | 699 (2.0%) |
|  | Benzoate degradation via CoA ligation | 6891 (19.5%) |
|  | Benzoate degradation | 1790 (5.1%) |
|  | Atrazine degradation | 1021 (2.9%) |
|  | Bisphenol degradation | 50 (0.1%) |
|  | Polycyclic aromatic hydrocarbon degradation | 1606 (4.5%) |
|  | Nitrotoluene degradation | 2585 (7.3%) |
|  | Geraniol degradation | 2180 (6.2%) |
|  | Fluorobenzoate degradation | 554 (1.6%) |
|  | Metabolism of xenobiotics by cytochrome P450 | 1476 (4.2%) |
|  | Drug metabolism - cytochrome P450 | 1596 (4.5%) |
|  | Drug metabolism - other enzymes | 3897 (11.0%) |

**Additional file 6: Table S2.** The relative number of genes assigned to each biological pathway (cont.).

| **Biological pathway** | **Pathway** | **MPI** |
| --- | --- | --- |
| Transcription | RNA polymerase | 8462 (87.4%) |
|  | Basal transcription factors | 1178 (12.2%) |
|  | Spliceosome | 47 (0.5%) |
| Translation | Ribosome | 15295 (35.6%) |
|  | Aminoacyl-tRNA biosynthesis | 27681 (64.4%) |
| Folding, sorting and degradation | Protein export | 7230 (38.1%) |
|  | SNARE interactions in vesicular transport | 3 (0.0%) |
|  | Ubiquitin mediated proteolysis | 19 (0.1%) |
|  | Proteasome | 1558 (8.2%) |
|  | RNA degradation | 10184 (53.6%) |
| Replication and repair | DNA replication | 8897 (19.1%) |
|  | Base excision repair | 6375 (13.7%) |
|  | Nucleotide excision repair | 10804 (23.2%) |
|  | Mismatch repair | 9713 (20.8%) |
|  | Homologous recombination | 9362 (20.1%) |
|  | Non-homologous end-joining | 1508 (3.2%) |
| Membrane transport | ABC transporters | 34694 (80.5%) |
|  | Phosphotransferase system (PTS) | 618 (1.4%) |
|  | Bacterial secretion system | 7781 (18.1%) |
| Signal transduction | Two-component system | 17265 (94.8%) |
|  | MAPK signaling pathway | 8 (0.0%) |
|  | MAPK signaling pathway - fly | 3 (0.0%) |
|  | MAPK signaling pathway - yeast | 121 (0.7%) |
|  | ErbB signaling pathway | 11 (0.1%) |
|  | Wnt signaling pathway | 6 (0.0%) |
|  | Notch signaling pathway | 2 (0.0%) |
|  | Hedgehog signaling pathway | 1 (0.0%) |
|  | TGF-beta signaling pathway | 3 (0.0%) |
|  | VEGF signaling pathway | 11 (0.1%) |
|  | Jak-STAT signaling pathway | 3 (0.0%) |
|  | Calcium signaling pathway | 7 (0.0%) |
|  | Phosphatidylinositol signaling system | 705 (3.9%) |
|  | mTOR signaling pathway | 59 (0.3%) |
| Signaling molecules and interaction | Neuroactive ligand-receptor interaction | 0 (0.0%) |
|  | Cytokine-cytokine receptor interaction | 0 (0.0%) |
|  | ECM-receptor interaction | 2 (100.0%) |
| Transport and catabolism | KEndocytosis | 12 (0.1%) |
|  | Lysosome | 1067 (10.1%) |
|  | Peroxisome | 4719 (44.8%) |
|  | Regulation of autophagy | 3 (0.0%) |
|  | Bacterial chemotaxis | 2865 (27.2%) |
|  | Flagellar assembly | 1854 (17.6%) |
|  | Regulation of actin cytoskeleton | 12 (0.1%) |
| Cell growth and death | Cell cycle | 26 (0.4%) |
|  | Cell cycle - yeast | 18 (0.3%) |
|  | Cell cycle - Caulobacter | 6531 (94.1%) |
|  | Meiosis - yeast | 216 (3.1%) |
|  | Oocyte meiosis | 6 (0.1%) |
|  | Apoptosis | 89 (1.3%) |
|  | signaling pathway | 51 (0.7%) |

**Additional file 6: Table S2.** The relative number of genes assigned to each biological pathway (cont.).

| **Biological pathway** | **Pathway** | **MPI** |
| --- | --- | --- |
| Cellular community - eukaryotes | Focal adhesion | 8 (34.8%) |
|  | Adherens junction | 2 (8.7%) |
|  | Tight junction | 7 (30.4%) |
|  | Gap junction | 6 (26.1%) |
| Immune system | Hematopoietic cell lineage | 2 (0.0%) |
|  | Complement and coagulation cascades | 0 (0.0%) |
|  | Toll-like receptor signaling pathway | 3 (0.1%) |
|  | NOD-like receptor signaling pathway | 521 (10.5%) |
|  | RIG-I-like receptor signaling pathway | 56 (1.1%) |
|  | Cytosolic DNA-sensing pathway | 0 (0.0%) |
|  | Natural killer cell mediated cytotoxicity | 8 (0.2%) |
|  | Antigen processing and presentation | 522 (10.5%) |
|  | T cell receptor signaling pathway | 7 (0.1%) |
|  | B cell receptor signaling pathway | 7 (0.1%) |
|  | Fc epsilon RI signaling pathway | 6 (0.1%) |
|  | Fc gamma R-mediated phagocytosis | 8 (0.2%) |
|  | Leukocyte transendothelial migration | 8 (0.2%) |
|  | Chemokine signaling pathway | 6 (0.1%) |
|  | Insulin signaling pathway | 1447 (29.1%) |
|  | Adipocytokine signaling pathway | 2366 (47.6%) |
| Endocrine system | PPAR signaling pathway | 3850 (84.0%) |
|  | GnRH signaling pathway | 7 (0.2%) |
|  | Progesterone-mediated oocyte maturation | 528 (11.5%) |
|  | Melanogenesis | 49 (1.1%) |
|  | Renin-angiotensin system | 151 (3.3%) |
| Circulatory system | Cardiac muscle contraction | 378 (84.2%) |
|  | Vascular smooth muscle contraction | 3 (0.7%) |
|  | Aldosterone-regulated sodium reabsorption | 7 (1.6%) |
|  | Vasopressin-regulated water reabsorption | 61 (13.6%) |
| Excretory system | Proximal tubule bicarbonate reclamation | 1609 (100.0%) |
| Nervous system | Long-term potentiation | 7 (31.8%) |
|  | Long-term depression | 7 (31.8%) |
|  | Neurotrophin signaling pathway | 8 (36.4%) |
| Sensory system | Olfactory transduction | 4 (100.0%) |
|  | Taste transduction | 0 (0.0%) |
| Development | Dorso-ventral axis formation | 6 (60.0%) |
|  | Axon guidance | 4 (40.0%) |
| Environmental adaptation | Circadian rhythm | 1 (0.0%) |
|  | Circadian rhythm - plant | 0 (0.0%) |
|  | Plant-pathogen interaction | 3392 (100.0%) |
| Cancers: Specific types | Colorectal cancer | 60 (4.5%) |
|  | Pancreatic cancer | 52 (3.9%) |
|  | Glioma | 12 (0.9%) |
|  | Thyroid cancer | 3 (0.2%) |
|  | Acute myeloid leukemia | 10 (0.8%) |
|  | Chronic myeloid leukemia | 8 (0.6%) |
|  | Basal cell carcinoma | 1 (0.1%) |
|  | *Melanoma* | 6 (0.5%) |
|  | Renal cell carcinoma | 459 (34.6%) |

**Additional file 6: Table S2.** The relative number of genes assigned to each biological pathway (cont.).

| **Biological pathway** | **Pathway** | **MPI** |
| --- | --- | --- |
| Cancers: Specific types | Bladder cancer | 116 (8.8%) |
|  | Prostate cancer | 530 (40.0%) |
|  | Endometrial cancer | 6 (0.5%) |
|  | Small cell lung cancer | 55 (4.2%) |
|  | Non-small cell lung cancer | 7 (0.5%) |
| Immune diseases | Systemic lupus erythematosus | 27 (8.0%) |
|  | Primary immunodeficiency | 312 (92.0%) |
| Neurodegenerative diseases | Alzheimer's disease | 1327 (37.6%) |
|  | Parkinson's disease | 476 (13.5%) |
|  | Amyotrophic lateral sclerosis (ALS) | 568 (16.1%) |
|  | Huntington's disease | 1122 (31.8%) |
|  | Prion diseases | 40 (1.1%) |
| Cardiovascular diseases | Hypertrophic cardiomyopathy (HCM) | 182 (56.7%) |
|  | Arrhythmogenic right ventricular cardiomyopathy (ARVC) | 3 (0.9%) |
|  | Dilated cardiomyopathy (DCM) | 84 (26.2%) |
|  | Viral myocarditis | 52 (16.2%) |
| Endocrine and metabolic diseases | Type I diabetes mellitus | 1724 (75.5%) |
|  | Type II diabetes mellitus | 558 (24.5%) |
|  | Maturity onset diabetes of the young | 0 (0.0%) |
| Infectious diseases: Bacterial | *Vibrio cholerae* infection | 67 (4.0%) |
|  | *Vibrio cholerae* pathogenic cycle | 856 (50.9%) |
|  | Epithelial cell signaling in Helicobacter pylori infection | 750 (44.6%) |
|  | Pathogenic *Escherichia coli* infection | 5 (0.3%) |
|  | Leishmaniasis | 3 (0.2%) |
