# Supplementary material for: Shotgun metagenomic sequencing from Manao-Pee cave, Thailand, reveals insight into the microbial community structure and its metabolic potential
Source: BMC Microbiol. 2019 Jun 27;19:144. doi: 10.1186/s12866-019-1521-8 (PMC6598295; doi:10.1186/s12866-019-1521-8)
Supplement: Supplementary file 7 — Table S3. The identified microbial genes involved in the oxidative phosphorylation pathway. (DOCX 17 kb) [file 12866_2019_1521_MOESM7_ESM.docx]

**Additional file 7: Table S3.** The identified microbial genes involved in the oxidative phosphorylation pathway.

| **Enzyme** | **The number of reads** |
| --- | --- |
| K00234 succinate dehydrogenase (ubiquinone) flavoprotein subunit [EC:1.3.5.1] | 4 |
| K00235 succinate dehydrogenase (ubiquinone) iron-sulfur subunit [EC:1.3.5.1] | 5 |
| K00239 succinate dehydrogenase / fumarate reductase, flavoprotein subunit [EC:1.3.5.1 1.3.5.4] | 1781 |
| K00240 succinate dehydrogenase / fumarate reductase, iron-sulfur subunit [EC:1.3.5.1 1.3.5.4] | 580 |
| K00241 succinate dehydrogenase / fumarate reductase, cytochrome b subunit | 194 |
| K00242 succinate dehydrogenase / fumarate reductase, membrane anchor subunit | 167 |
| K00244 fumarate reductase flavoprotein subunit [EC:1.3.5.4] | 56 |
| K00245 fumarate reductase iron-sulfur subunit [EC:1.3.5.4] | 12 |
| K00246 fumarate reductase subunit C | 4 |
| K00234 succinate dehydrogenase (ubiquinone) flavoprotein subunit [EC:1.3.5.1] | 4 |
| K00235 succinate dehydrogenase (ubiquinone) iron-sulfur subunit [EC:1.3.5.1] | 5 |
| K00239 succinate dehydrogenase / fumarate reductase, flavoprotein subunit [EC:1.3.5.1 1.3.5.4] | 1781 |
| K00240 succinate dehydrogenase / fumarate reductase, iron-sulfur subunit [EC:1.3.5.1 1.3.5.4] | 580 |
| K00241 succinate dehydrogenase / fumarate reductase, cytochrome b subunit | 194 |
| K00242 succinate dehydrogenase / fumarate reductase, membrane anchor subunit | 167 |
| K00244 fumarate reductase flavoprotein subunit [EC:1.3.5.4] | 56 |
| K00245 fumarate reductase iron-sulfur subunit [EC:1.3.5.4] | 12 |
| K00246 fumarate reductase subunit C | 4 |
| K00339 NADH-quinone oxidoreductase subunit J [EC:1.6.5.3] | 392 |
| K00340 NADH-quinone oxidoreductase subunit K [EC:1.6.5.3] | 414 |
| K00341 NADH-quinone oxidoreductase subunit L [EC:1.6.5.3] | 1509 |
| K00342 NADH-quinone oxidoreductase subunit M [EC:1.6.5.3] | 1016 |
| K00343 NADH-quinone oxidoreductase subunit N [EC:1.6.5.3] | 873 |
| K00356 NADH dehydrogenase [EC:1.6.99.3] | 105 |
| K00404 cytochrome c oxidase cbb3-type subunit I [EC:1.9.3.1] | 28 |
| K00405 cytochrome c oxidase cbb3-type subunit II | 38 |
| K00406 cytochrome c oxidase cbb3-type subunit III | 18 |
| K00411 ubiquinol-cytochrome c reductase iron-sulfur subunit [EC:1.10.2.2] | 20 |
| K00412 ubiquinol-cytochrome c reductase cytochrome b subunit | 318 |
| K00413 ubiquinol-cytochrome c reductase cytochrome c1 subunit | 32 |
| K00425 cytochrome d ubiquinol oxidase subunit I [EC:1.10.3.-] | 352 |
| K00426 cytochrome d ubiquinol oxidase subunit II [EC:1.10.3.-] | 185 |
| K00937 polyphosphate kinase [EC:2.7.4.1] | 499 |
| K01507 inorganic pyrophosphatase [EC:3.6.1.1] | 594 |
| K02108 F-type H+-transporting ATPase subunit a | 328 |
| K02109 F-type H+-transporting ATPase subunit b | 160 |
| K02110 F-type H+-transporting ATPase subunit c | 99 |
| K02111 F-type H+-transporting ATPase subunit alpha [EC:3.6.3.14] | 1228 |

**Additional file 7: Table S3.** The identified microbial genes involved in the oxidative phosphorylation pathway (cont.)

| **Enzyme** | **The number of reads** |
| --- | --- |
| K02112 F-type H+-transporting ATPase subunit beta [EC:3.6.3.14] | 1058 |
| K02113 F-type H+-transporting ATPase subunit delta | 128 |
| K02114 F-type H+-transporting ATPase subunit epsilon | 137 |
| K02115 F-type H+-transporting ATPase subunit gamma | 466 |
| K02117 V/A-type H+-transporting ATPase subunit A [EC:3.6.3.14] | 420 |
| K02118 V/A-type H+-transporting ATPase subunit B | 334 |
| K02119 V/A-type H+-transporting ATPase subunit C | 239 |
| K02120 V/A-type H+-transporting ATPase subunit D | 253 |
| K02121 V/A-type H+-transporting ATPase subunit E | 104 |
| K02122 V/A-type H+-transporting ATPase subunit F | 62 |
| K02123 V/A-type H+-transporting ATPase subunit I | 590 |
| K02124 V/A-type H+-transporting ATPase subunit K | 68 |
| K02126 F-type H+-transporting ATPase subunit a | 5 |
| K02132 F-type H+-transporting ATPase subunit alpha | 8 |
| K02133 F-type H+-transporting ATPase subunit beta [EC:3.6.3.14] | 3 |
| K02144 V-type H+-transporting ATPase subunit H | 2 |
| K02145 V-type H+-transporting ATPase subunit A [EC:3.6.3.14] | 18 |
| K02257 protoheme IX farnesyltransferase [EC:2.5.1.-] | 1 |
| K02258 cytochrome c oxidase assembly protein subunit 11 | 5 |
| K02259 cytochrome c oxidase assembly protein subunit 15 | 245 |
| K02261 cytochrome c oxidase subunit 2 | 1 |
| K02262 cytochrome c oxidase subunit 3 | 6 |
| K02274 cytochrome c oxidase subunit I [EC:1.9.3.1] | 2012 |
| K02275 cytochrome c oxidase subunit II [EC:1.9.3.1] | 567 |
| K02276 cytochrome c oxidase subunit III [EC:1.9.3.1] | 339 |
| K02277 cytochrome c oxidase subunit IV [EC:1.9.3.1] | 34 |
| K02297 cytochrome o ubiquinol oxidase subunit II [EC:1.10.3.-] | 114 |
| K02298 cytochrome o ubiquinol oxidase subunit I [EC:1.10.3.-] | 314 |
| K02299 cytochrome o ubiquinol oxidase subunit III [EC:1.10.3.-] | 40 |
| K02300 cytochrome o ubiquinol oxidase operon protein cyoD | 37 |
| K02301 protoheme IX farnesyltransferase [EC:2.5.1.-] | 553 |
| K03883 NADH-ubiquinone oxidoreductase chain 5 [EC:1.6.5.3] | 1 |
| K03885 NADH dehydrogenase [EC:1.6.99.3] | 645 |
| K03887 menaquinol-cytochrome c reductase cytochrome b subunit | 49 |
| K03888 menaquinol-cytochrome c reductase cytochrome b/c subunit | 19 |
| K03889 ubiquinol-cytochrome c reductase cytochrome c subunit | 105 |
| K03890 ubiquinol-cytochrome c reductase iron-sulfur subunit | 185 |
| K03891 ubiquinol-cytochrome c reductase cytochrome b subunit | 421 |
| K03935 NADH dehydrogenase (ubiquinone) Fe-S protein 2 [EC:1.6.5.3 1.6.99.3] | 5 |
| K03940 NADH dehydrogenase (ubiquinone) Fe-S protein 7 [EC:1.6.5.3 1.6.99.3] | 6 |
| K03941 NADH dehydrogenase (ubiquinone) Fe-S protein 8 [EC:1.6.5.3 1.6.99.3] | 1 |
| K03942 NADH dehydrogenase (ubiquinone) flavoprotein 1 [EC:1.6.5.3 1.6.99.3] | 1 |

**Additional file 7: Table S3.** The identified microbial genes involved in the oxidative phosphorylation pathway (cont.)

| **Enzyme** | **The number of reads** |
| --- | --- |
| K03943 NADH dehydrogenase (ubiquinone) flavoprotein 2 [EC:1.6.5.3 1.6.99.3] | 4 |
| K03955 NADH dehydrogenase (ubiquinone) 1 alpha/beta subcomplex 1 | 1 |
| K05574 NAD(P)H-quinone oxidoreductase subunit 3 [EC:1.6.5.3] | 1 |
| K05575 NAD(P)H-quinone oxidoreductase subunit 4 [EC:1.6.5.3] | 4 |
| K05576 NAD(P)H-quinone oxidoreductase subunit 4L [EC:1.6.5.3] | 1 |
| K05577 NAD(P)H-quinone oxidoreductase subunit 5 [EC:1.6.5.3] | 26 |
| K05578 NAD(P)H-quinone oxidoreductase subunit 6 [EC:1.6.5.3] | 15 |
| K05579 NAD(P)H-quinone oxidoreductase subunit H [EC:1.6.5.3] | 1 |
| K05586 bidirectional [NiFe] hydrogenase diaphorase subunit [EC:1.6.5.3] | 1 |
| K05587 bidirectional [NiFe] hydrogenase diaphorase subunit [EC:1.6.5.3] | 1 |
| K05588 bidirectional [NiFe] hydrogenase diaphorase subunit [EC:1.6.5.3] | 3 |
| K05903 NADH dehydrogenase (quinone) [EC:1.6.99.5] | 89 |
| K06019 pyrophosphatase PpaX [EC:3.6.1.1] | 7 |
| K13378 NADH-quinone oxidoreductase subunit C/D [EC:1.6.5.3] | 75 |
