# Supplementary material for: Shotgun metagenomic sequencing from Manao-Pee cave, Thailand, reveals insight into the microbial community structure and its metabolic potential
Source: BMC Microbiol. 2019 Jun 27;19:144. doi: 10.1186/s12866-019-1521-8 (PMC6598295; doi:10.1186/s12866-019-1521-8)
Supplement: Supplementary file 8 — Table S4. The identified microbial genes involved in methane metabolism pathway. (DOCX 16 kb) [file 12866_2019_1521_MOESM8_ESM.docx]

**Additional file 8: Table S4.** The identified microbial genes involved in methane metabolism pathway.

| **Enzyme** | **The number of reads** |
| --- | --- |
| K00018 glycerate dehydrogenase [EC:1.1.1.29] | 69 |
| K00024 malate dehydrogenase [EC:1.1.1.37] | 610 |
| K00058 D-3-phosphoglycerate dehydrogenase [EC:1.1.1.95] | 1148 |
| K00121 S-(hydroxymethyl)glutathione dehydrogenase / alcohol dehydrogenase [EC:1.1.1.284 1.1.1.1] | 395 |
| K00122 formate dehydrogenase [EC:1.2.1.2] | 134 |
| K00123 formate dehydrogenase major subunit [EC:1.2.1.2] | 1368 |
| K00124 formate dehydrogenase iron-sulfur subunit | 264 |
| K00127 formate dehydrogenase subunit gamma | 130 |
| K00148 glutathione-independent formaldehyde dehydrogenase [EC:1.2.1.46] | 126 |
| K00169 pyruvate ferredoxin oxidoreductase, alpha subunit [EC:1.2.7.1] | 121 |
| K00170 pyruvate ferredoxin oxidoreductase, beta subunit [EC:1.2.7.1] | 181 |
| K00171 pyruvate ferredoxin oxidoreductase, delta subunit [EC:1.2.7.1] | 47 |
| K00172 pyruvate ferredoxin oxidoreductase, gamma subunit [EC:1.2.7.1] | 30 |
| K00200 formylmethanofuran dehydrogenase subunit A [EC:1.2.99.5] | 18 |
| K00201 formylmethanofuran dehydrogenase subunit B [EC:1.2.99.5] | 2 |
| K00202 formylmethanofuran dehydrogenase subunit C [EC:1.2.99.5] | 2 |
| K00297 methylenetetrahydrofolate reductase (NADPH) [EC:1.5.1.20] | 284 |
| K00317 dimethylamine/trimethylamine dehydrogenase [EC:1.5.8.1 1.5.8.2] | 48 |
| K00320 5,10-methylenetetrahydromethanopterin reductase [EC:1.5.98.2] | 79 |
| K00441 coenzyme F420 hydrogenase subunit beta [EC:1.12.98.1] | 57 |
| K00485 dimethylaniline monooxygenase (N-oxide forming) [EC:1.14.13.8] | 2 |
| K00577 tetrahydromethanopterin S-methyltransferase subunit A [EC:2.1.1.86] | 56 |
| K00600 glycine hydroxymethyltransferase [EC:2.1.2.1] | 942 |
| K00625 phosphate acetyltransferase [EC:2.3.1.8] | 36 |
| K00672 formylmethanofuran--tetrahydromethanopterin N-formyltransferase [EC:2.3.1.101] | 4 |
| K00831 phosphoserine aminotransferase [EC:2.6.1.52] | 337 |
| K00850 6-phosphofructokinase 1 [EC:2.7.1.11] | 534 |
| K00863 dihydroxyacetone kinase [EC:2.7.1.29] | 54 |
| K00865 glycerate kinase [EC:2.7.1.31] | 55 |
| K00925 acetate kinase [EC:2.7.2.1] | 118 |
| K01007 pyruvate, water dikinase [EC:2.7.9.2] | 753 |
| K01070 S-formylglutathione hydrolase [EC:3.1.2.12] | 21 |
| K01079 phosphoserine phosphatase [EC:3.1.3.3] | 298 |
| K01499 methenyltetrahydromethanopterin cyclohydrolase [EC:3.5.4.27] | 4 |
| K01595 phosphoenolpyruvate carboxylase [EC:4.1.1.31] | 361 |
| K01621 phosphoketolase [EC:4.1.2.9] | 104 |
| K01624 fructose-bisphosphate aldolase, class II [EC:4.1.2.13] | 290 |
| K01689 enolase [EC:4.2.1.11] | 1098 |
| K01834 2,3-bisphosphoglycerate-dependent phosphoglycerate mutase [EC:5.4.2.11] | 196 |
| K01895 acetyl-CoA synthetase [EC:6.2.1.1] | 2424 |
| K02117 V/A-type H+-transporting ATPase subunit A [EC:3.6.3.14] | 420 |

**Additional file 8: Table S4.** The identified microbial genes involved in the methane metabolism pathway (cont.)

| **Enzyme** | **The number of reads** |
| --- | --- |
| K02118 V/A-type H+-transporting ATPase subunit B | 334 |
| K02119 V/A-type H+-transporting ATPase subunit C | 239 |
| K02120 V/A-type H+-transporting ATPase subunit D | 253 |
| K02121 V/A-type H+-transporting ATPase subunit E | 104 |
| K02122 V/A-type H+-transporting ATPase subunit F | 62 |
| K02123 V/A-type H+-transporting ATPase subunit I | 590 |
| K02124 V/A-type H+-transporting ATPase subunit K | 68 |
| K02203 phosphoserine / homoserine phosphotransferase [EC:3.1.3.3 2.7.1.39] | 3 |
| K03313 | 122 |
| K03314 | 3 |
| K03315 | 6 |
| K03388 heterodisulfide reductase subunit A [EC:1.8.98.1] | 5 |
| K03389 heterodisulfide reductase subunit B [EC:1.8.98.1] | 3 |
| K03390 heterodisulfide reductase subunit C [EC:1.8.98.1] | 1 |
| K03518 carbon-monoxide dehydrogenase small subunit [EC:1.2.99.2] | 332 |
| K03519 carbon-monoxide dehydrogenase medium subunit [EC:1.2.99.2] | 393 |
| K03520 carbon-monoxide dehydrogenase large subunit [EC:1.2.99.2] | 1090 |
| K03781 catalase [EC:1.11.1.6] | 479 |
| K03782 catalase-peroxidase [EC:1.11.1.21] | 270 |
| K03841 fructose-1,6-bisphosphatase I [EC:3.1.3.11] | 152 |
| K05884 L-2-hydroxycarboxylate dehydrogenase (NAD+) [EC:1.1.1.337] | 1 |
| K05979 2-phosphosulfolactate phosphatase [EC:3.1.3.71] | 89 |
| K06034 sulfopyruvate decarboxylase subunit alpha [EC:4.1.1.79] | 86 |
| K06862 | 40 |
| K08094 6-phospho-3-hexuloisomerase [EC:5.3.1.27] | 2 |
| K08097 phosphosulfolactate synthase [EC:4.4.1.19] | 341 |
| K08685 quinohemoprotein amine dehydrogenase [EC:1.4.9.1] | 3 |
| K08691 malyl-CoA/(S)-citramalyl-CoA lyase [EC:4.1.3.24 4.1.3.25] | 52 |
| K08692 malate-CoA ligase subunit alpha [EC:6.2.1.9] | 6 |
| K10713 5,6,7,8-tetrahydromethanopterin hydro-lyase [EC:4.2.1.147] | 8 |
| K11261 formylmethanofuran dehydrogenase subunit E [EC:1.2.99.5] | 7 |
| K13039 sulfopyruvate decarboxylase subunit beta [EC:4.1.1.79] | 6 |
| K13788 phosphate acetyltransferase [EC:2.3.1.8] | 70 |
| K13831 3-hexulose-6-phosphate synthase / 6-phospho-3-hexuloisomerase [EC:4.1.2.43 5.3.1.27] | 3 |
| K14083 trimethylamine---corrinoid protein Co-methyltransferase [EC:2.1.1.250] | 4 |
| K10944 methane/ammonia monooxygenase subunit A [EC:1.14.18.3 1.14.99.39] (membrane-bound) | 15 |
| K10945 methane/ammonia monooxygenase subunit B | 3 |
| K10946 methane/ammonia monooxygenase subunit C | 237 |
