# Supplementary material for: Shotgun metagenomic sequencing from Manao-Pee cave, Thailand, reveals insight into the microbial community structure and its metabolic potential
Source: BMC Microbiol. 2019 Jun 27;19:144. doi: 10.1186/s12866-019-1521-8 (PMC6598295; doi:10.1186/s12866-019-1521-8)
Supplement: Supplementary file 9 — Table S5. The identified microbial genes involved in carbon fixation pathways in prokaryotes. (DOCX 15 kb) [file 12866_2019_1521_MOESM9_ESM.docx]

| **Enzyme** | **The number of reads** |
| --- | --- |
| K00024 malate dehydrogenase [EC:1.1.1.37] | 610 |
| K00031 isocitrate dehydrogenase [EC:1.1.1.42] | 1024 |
| K00169 pyruvate ferredoxin oxidoreductase, alpha subunit [EC:1.2.7.1] | 121 |
| K00170 pyruvate ferredoxin oxidoreductase, beta subunit [EC:1.2.7.1] | 181 |
| K00171 pyruvate ferredoxin oxidoreductase, delta subunit [EC:1.2.7.1] | 47 |
| K00172 pyruvate ferredoxin oxidoreductase, gamma subunit [EC:1.2.7.1] | 30 |
| K00174 2-oxoglutarate ferredoxin oxidoreductase subunit alpha [EC:1.2.7.3] | 1009 |
| K00175 2-oxoglutarate ferredoxin oxidoreductase subunit beta [EC:1.2.7.3] | 540 |
| K00176 2-oxoglutarate ferredoxin oxidoreductase subunit delta [EC:1.2.7.3] | 2 |
| K00177 2-oxoglutarate ferredoxin oxidoreductase subunit gamma [EC:1.2.7.3] | 2 |
| K00239 succinate dehydrogenase / fumarate reductase, flavoprotein subunit [EC:1.3.5.1 1.3.5.4] | 1781 |
| K00240 succinate dehydrogenase / fumarate reductase, iron-sulfur subunit [EC:1.3.5.1 1.3.5.4] | 580 |
| K00241 succinate dehydrogenase / fumarate reductase, cytochrome b subunit | 194 |
| K00242 succinate dehydrogenase / fumarate reductase, membrane anchor subunit | 167 |
| K00244 fumarate reductase flavoprotein subunit [EC:1.3.5.4] | 56 |
| K00245 fumarate reductase iron-sulfur subunit [EC:1.3.5.4] | 12 |
| K00246 fumarate reductase subunit C | 4 |
| K00259 alanine dehydrogenase [EC:1.4.1.1] | 575 |
| K01007 pyruvate, water dikinase [EC:2.7.9.2] | 753 |
| K01595 phosphoenolpyruvate carboxylase [EC:4.1.1.31] | 361 |
| K01676 fumarate hydratase, class I [EC:4.2.1.2] | 161 |
| K01677 fumarate hydratase subunit alpha [EC:4.2.1.2] | 65 |
| K01678 fumarate hydratase subunit beta [EC:4.2.1.2] | 28 |
| K01679 fumarate hydratase, class II [EC:4.2.1.2] | 453 |
| K01681 aconitate hydratase [EC:4.2.1.3] | 1838 |
| K01682 aconitate hydratase 2 / 2-methylisocitrate dehydratase [EC:4.2.1.3 4.2.1.99] | 2 |
| K01895 acetyl-CoA synthetase [EC:6.2.1.1] | 2424 |
| K01902 succinyl-CoA synthetase alpha subunit [EC:6.2.1.5] | 609 |
| K01903 succinyl-CoA synthetase beta subunit [EC:6.2.1.5] | 769 |
| K00024 malate dehydrogenase [EC:1.1.1.37] | 610 |
| K00031 isocitrate dehydrogenase [EC:1.1.1.42] | 1024 |
| K00169 pyruvate ferredoxin oxidoreductase, alpha subunit [EC:1.2.7.1] | 121 |
| K00170 pyruvate ferredoxin oxidoreductase, beta subunit [EC:1.2.7.1] | 181 |
| K00171 pyruvate ferredoxin oxidoreductase, delta subunit [EC:1.2.7.1] | 47 |
| K00172 pyruvate ferredoxin oxidoreductase, gamma subunit [EC:1.2.7.1] | 30 |
| K00174 2-oxoglutarate ferredoxin oxidoreductase subunit alpha [EC:1.2.7.3] | 1009 |
| K00175 2-oxoglutarate ferredoxin oxidoreductase subunit beta [EC:1.2.7.3] | 540 |
| K00176 2-oxoglutarate ferredoxin oxidoreductase subunit delta [EC:1.2.7.3] | 2 |
| K00177 2-oxoglutarate ferredoxin oxidoreductase subunit gamma [EC:1.2.7.3] | 2 |
| K00239 succinate dehydrogenase / fumarate reductase, flavoprotein subunit [EC:1.3.5.1 1.3.5.4] | 1781 |
| K00240 succinate dehydrogenase / fumarate reductase, iron-sulfur subunit [EC:1.3.5.1 1.3.5.4] | 580 |

**Additional file 9: Table S5.** The identified microbial genes involved in carbon fixation pathways in prokaryotes.
