# Supplementary material for: Shotgun metagenomic sequencing from Manao-Pee cave, Thailand, reveals insight into the microbial community structure and its metabolic potential
Source: BMC Microbiol. 2019 Jun 27;19:144. doi: 10.1186/s12866-019-1521-8 (PMC6598295; doi:10.1186/s12866-019-1521-8)
Supplement: Supplementary file 10 — Table S6. The identified microbial genes involved in carbon fixation from photosynthetic organisms. (DOCX 14 kb) [file 12866_2019_1521_MOESM10_ESM.docx]

| **Enzyme** | **The number of reads** |
| --- | --- |
| K00024 malate dehydrogenase [EC:1.1.1.37] | 610 |
| K00029 malate dehydrogenase (oxaloacetate-decarboxylating)(NADP+) [EC:1.1.1.40] | 106 |
| K00150 glyceraldehyde-3-phosphate dehydrogenase (NAD(P)) [EC:1.2.1.59] | 228 |
| K00615 transketolase [EC:2.2.1.1] | 1633 |
| K00812 aspartate aminotransferase [EC:2.6.1.1] | 566 |
| K00813 aspartate aminotransferase [EC:2.6.1.1] | 23 |
| K00855 phosphoribulokinase [EC:2.7.1.19] | 77 |
| K00873 pyruvate kinase [EC:2.7.1.40] | 552 |
| K00927 phosphoglycerate kinase [EC:2.7.2.3] | 613 |
| K01006 pyruvate, orthophosphate dikinase [EC:2.7.9.1] | 1286 |
| K01595 phosphoenolpyruvate carboxylase [EC:4.1.1.31] | 361 |
| K01601 ribulose-bisphosphate carboxylase large chain [EC:4.1.1.39] | 59 |
| K01602 ribulose-bisphosphate carboxylase small chain [EC:4.1.1.39] | 6 |
| K01610 phosphoenolpyruvate carboxykinase (ATP) [EC:4.1.1.49] | 557 |
| K01621 phosphoketolase [EC:4.1.2.9] | 104 |
| K01623 fructose-bisphosphate aldolase, class I [EC:4.1.2.13] | 180 |
| K01624 fructose-bisphosphate aldolase, class II [EC:4.1.2.13] | 290 |
| K01632 fructose-6-phosphate phosphoketolase [EC:4.1.2.22] | 20 |
| K01783 ribulose-phosphate 3-epimerase [EC:5.1.3.1] | 215 |
| K01803 triosephosphate isomerase (TIM) [EC:5.3.1.1] | 425 |
| K01807 ribose 5-phosphate isomerase A [EC:5.3.1.6] | 281 |
| K01808 ribose 5-phosphate isomerase B [EC:5.3.1.6] | 113 |
| K02446 fructose-1,6-bisphosphatase II [EC:3.1.3.11] | 257 |
| K03841 fructose-1,6-bisphosphatase I [EC:3.1.3.11] | 152 |
| K04041 fructose-1,6-bisphosphatase III [EC:3.1.3.11] | 11 |
| K11358 aspartate aminotransferase [EC:2.6.1.1] | 27 |
| K11532 fructose-1,6-bisphosphatase II / sedoheptulose-1,7-bisphosphatase [EC:3.1.3.11 3.1.3.37] | 4 |

**Additional file 10: Table S6.** The identified microbial genes involved in carbon fixation from photosynthetic organisms.
