# Supplementary material for: Shotgun metagenomic sequencing from Manao-Pee cave, Thailand, reveals insight into the microbial community structure and its metabolic potential
Source: BMC Microbiol. 2019 Jun 27;19:144. doi: 10.1186/s12866-019-1521-8 (PMC6598295; doi:10.1186/s12866-019-1521-8)
Supplement: Supplementary file 11 — Table S7. The identified microbial genes involved in the photosynthetic pathway. (DOCX 14 kb) [file 12866_2019_1521_MOESM11_ESM.docx]

| **Enzyme** | **The number of reads** |
| --- | --- |
| K02108 F-type H+-transporting ATPase subunit a | 328 |
| K02109 F-type H+-transporting ATPase subunit b | 160 |
| K02110 F-type H+-transporting ATPase subunit c | 99 |
| K02111 F-type H+-transporting ATPase subunit alpha [EC:3.6.3.14] | 1228 |
| K02112 F-type H+-transporting ATPase subunit beta [EC:3.6.3.14] | 1058 |
| K02113 F-type H+-transporting ATPase subunit delta | 128 |
| K02114 F-type H+-transporting ATPase subunit epsilon | 137 |
| K02115 F-type H+-transporting ATPase subunit gamma | 466 |
| K02635 cytochrome b6 | 6 |
| K02636 cytochrome b6-f complex iron-sulfur subunit [EC:1.10.9.1] | 24 |
| K02639 ferredoxin | 7 |
| K02641 ferredoxin--NADP+ reductase [EC:1.18.1.2] | 1 |
| K02716 photosystem II oxygen-evolving enhancer protein 1 | 1 |

**Additional file 11: Table S7.** The identified microbial genes involved in the photosynthetic pathway.
