# Supplementary material for: Shotgun metagenomic sequencing from Manao-Pee cave, Thailand, reveals insight into the microbial community structure and its metabolic potential
Source: BMC Microbiol. 2019 Jun 27;19:144. doi: 10.1186/s12866-019-1521-8 (PMC6598295; doi:10.1186/s12866-019-1521-8)
Supplement: Supplementary file 12 — Table S8. The identified microbial genes involved in nitrogen metabolism pathway. (DOCX 16 kb) [file 12866_2019_1521_MOESM12_ESM.docx]

**Additional file 12: Table S8.** The identified microbial genes involved in nitrogen metabolism pathway.

| **Enzyme** | **The number of reads** |
| --- | --- |
| K00260 glutamate dehydrogenase [EC:1.4.1.2] | 10 |
| K00261 glutamate dehydrogenase (NAD(P)+) [EC:1.4.1.3] | 982 |
| K00262 glutamate dehydrogenase (NADP+) [EC:1.4.1.4] | 194 |
| K00264 glutamate synthase (NADPH/NADH) [EC:1.4.1.13 1.4.1.14] | 28 |
| K00265 glutamate synthase (NADPH/NADH) large chain [EC:1.4.1.13 1.4.1.14] | 1211 |
| K00266 glutamate synthase (NADPH/NADH) small chain [EC:1.4.1.13 1.4.1.14] | 424 |
| K00284 glutamate synthase (ferredoxin) [EC:1.4.7.1] | 641 |
| K00285 D-amino-acid dehydrogenase [EC:1.4.99.1] | 163 |
| K00360 assimilatory nitrate reductase electron transfer subunit [EC:1.7.99.4] | 8 |
| K00362 nitrite reductase (NADH) large subunit [EC:1.7.1.15] | 262 |
| K00363 nitrite reductase (NADH) small subunit [EC:1.7.1.15] | 77 |
| K00366 ferredoxin-nitrite reductase [EC:1.7.7.1] | 392 |
| K00367 ferredoxin-nitrate reductase [EC:1.7.7.2] | 13 |
| K00368 nitrite reductase (NO-forming) [EC:1.7.2.1] | 361 |
| K00370 nitrate reductase alpha subunit [EC:1.7.99.4] | 430 |
| K00371 nitrate reductase beta subunit [EC:1.7.99.4] | 156 |
| K00372 assimilatory nitrate reductase catalytic subunit [EC:1.7.99.4] | 180 |
| K00373 nitrate reductase delta subunit | 17 |
| K00374 nitrate reductase gamma subunit [EC:1.7.99.4] | 110 |
| K00376 nitrous-oxide reductase [EC:1.7.2.4] | 39 |
| K00459 nitronate monooxygenase [EC:1.13.12.16] | 105 |
| K00605 aminomethyltransferase [EC:2.1.2.10] | 843 |
| K00926 carbamate kinase [EC:2.7.2.2] | 88 |
| K01424 L-asparaginase [EC:3.5.1.1] | 142 |
| K01425 glutaminase [EC:3.5.1.2] | 19 |
| K01455 formamidase [EC:3.5.1.49] | 306 |
| K01501 nitrilase [EC:3.5.5.1] | 69 |
| K01667 tryptophanase [EC:4.1.99.1] | 12 |
| K01668 tyrosine phenol-lyase [EC:4.1.99.2] | 4 |
| K01672 carbonic anhydrase [EC:4.2.1.1] | 1 |
| K01673 carbonic anhydrase [EC:4.2.1.1] | 140 |
| K01674 carbonic anhydrase [EC:4.2.1.1] | 24 |
| K01725 cyanate lyase [EC:4.2.1.104] | 8 |
| K01744 aspartate ammonia-lyase [EC:4.3.1.1] | 262 |
| K01745 histidine ammonia-lyase [EC:4.3.1.3] | 405 |
| K01758 cystathionine gamma-lyase [EC:4.4.1.1] | 131 |
| K01760 cystathionine beta-lyase [EC:4.4.1.8] | 41 |
| K01914 aspartate--ammonia ligase [EC:6.3.1.1] | 6 |
| K01915 glutamine synthetase [EC:6.3.1.2] | 2391 |
| K01916 NAD+ synthase [EC:6.3.1.5] | 223 |
| K01953 asparagine synthase (glutamine-hydrolysing) [EC:6.3.5.4] | 1668 |

**Additional file 12: Table S8.** The identified microbial genes involved in nitrogen metabolism pathways (cont.)

| **Enzyme** | **The number of reads** |
| --- | --- |
| K02567 periplasmic nitrate reductase NapA [EC:1.7.99.4] | 12 |
| K03385 nitrite reductase (cytochrome c-552) [EC:1.7.2.2] | 13 |
| K04561 nitric oxide reductase subunit B [EC:1.7.2.5] | 105 |
| K04835 methylaspartate ammonia-lyase [EC:4.3.1.2] | 3 |
| K05597 glutamin-(asparagin-)ase [EC:3.5.1.38] | 1 |
| K10534 nitrate reductase (NAD(P)H) [EC:1.7.1.1 1.7.1.2 1.7.1.3] | 1 |
| K10535 hydroxylamine dehydrogenase [EC:1.7.2.6] | 5 |
| K10775 phenylalanine ammonia-lyase [EC:4.3.1.24] | 24 |
| K10944 methane/ammonia monooxygenase subunit A [EC:1.14.18.3 1.14.99.39] | 15 |
| K10945 methane/ammonia monooxygenase subunit B | 3 |
| K10946 methane/ammonia monooxygenase subunit C | 237 |
