# Supplementary material for: Shotgun metagenomic sequencing from Manao-Pee cave, Thailand, reveals insight into the microbial community structure and its metabolic potential
Source: BMC Microbiol. 2019 Jun 27;19:144. doi: 10.1186/s12866-019-1521-8 (PMC6598295; doi:10.1186/s12866-019-1521-8)
Supplement: Supplementary file 13 — Table S9. The identified microbial genes involved in sulfur metabolism pathway. (DOCX 15 kb) [file 12866_2019_1521_MOESM13_ESM.docx]

**Additional file 13: Table S9.** The identified microbial genes involved in sulfur metabolism pathway.

| **Enzyme** | **The number of reads** |
| --- | --- |
| K00380 sulfite reductase (NADPH) flavoprotein alpha-component [EC:1.8.1.2] | 145 |
| K00381 sulfite reductase (NADPH) hemoprotein beta-component [EC:1.8.1.2] | 427 |
| K00387 sulfite oxidase [EC:1.8.3.1] | 19 |
| K00390 phosphoadenosine phosphosulfate reductase [EC:1.8.4.8] | 542 |
| K00392 sulfite reductase (ferredoxin) [EC:1.8.7.1] | 636 |
| K00394 adenylylsulfate reductase, subunit A [EC:1.8.99.2] | 1 |
| K00640 serine O-acetyltransferase [EC:2.3.1.30] | 111 |
| K00641 homoserine O-acetyltransferase [EC:2.3.1.31] | 352 |
| K00651 homoserine O-succinyltransferase [EC:2.3.1.46] | 6 |
| K00860 adenylylsulfate kinase [EC:2.7.1.25] | 307 |
| K00955 bifunctional enzyme CysN/CysC [EC:2.7.7.4 2.7.1.25] | 140 |
| K00956 sulfate adenylyltransferase subunit 1 [EC:2.7.7.4] | 145 |
| K00957 sulfate adenylyltransferase subunit 2 [EC:2.7.7.4] | 306 |
| K00958 sulfate adenylyltransferase [EC:2.7.7.4] | 643 |
| K01014 aryl sulfotransferase [EC:2.8.2.1] | 3 |
| K01082 3'(2'), 5'-bisphosphate nucleotidase [EC:3.1.3.7] | 98 |
| K01738 cysteine synthase A [EC:2.5.1.47] | 496 |
| K01739 cystathionine gamma-synthase [EC:2.5.1.48] | 505 |
| K01760 cystathionine beta-lyase [EC:4.4.1.8] | 41 |
| K05301 sulfite dehydrogenase [EC:1.8.2.1] | 7 |
| K12339 cysteine synthase B [EC:2.5.1.47] | 723 |
| K13811 3'-phosphoadenosine 5'-phosphosulfate synthase [EC:2.7.7.4 2.7.1.25] | 1 |
