# Supplementary material for: Shotgun metagenomic sequencing from Manao-Pee cave, Thailand, reveals insight into the microbial community structure and its metabolic potential
Source: BMC Microbiol. 2019 Jun 27;19:144. doi: 10.1186/s12866-019-1521-8 (PMC6598295; doi:10.1186/s12866-019-1521-8)
Supplement: Supplementary file 14 — Figure S5. Comparison of bacterial diversity in the soil community of Manao-Pee cave at the phylum level by 16S rRNA sequencing versus shotgun metagenomic sequencing. Both techniques, at this taxonomic depth, gave comparable results, revealing that most Manao-Pee cave microbes belonged to the phyla Actinobacteria or Proteobacteria. (DOCX 950 kb) [file 12866_2019_1521_MOESM14_ESM.docx]

**
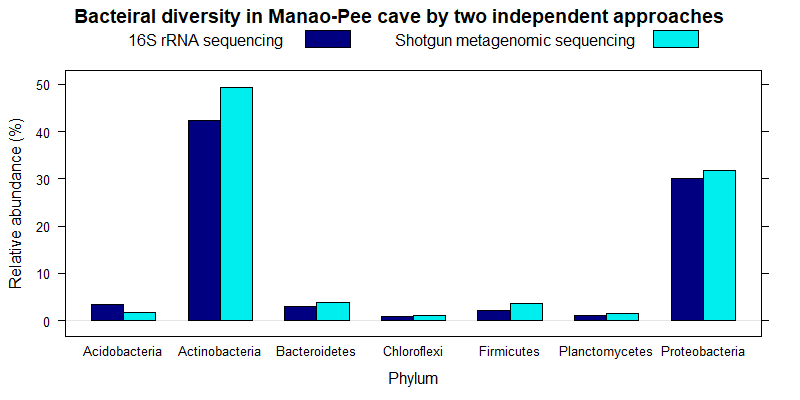
Additional file 14: Figure S5.** Comparison of bacterial diversity in the soil community of Manao-Pee cave at the phylum level by 16S rRNA sequencing versus shotgun metagenomic sequencing**.** Both techniques, at this taxonomic depth, gave comparable results, revealing that most Manao-Pee cave microbes belonged to the phyla *Actinobacteria* or *Proteobacteria*.
