# Supplementary material for: Shotgun metagenomic sequencing from Manao-Pee cave, Thailand, reveals insight into the microbial community structure and its metabolic potential
Source: BMC Microbiol. 2019 Jun 27;19:144. doi: 10.1186/s12866-019-1521-8 (PMC6598295; doi:10.1186/s12866-019-1521-8)
Supplement: Supplementary file 15 — Figure S6. Comparison of bacterial diversity in the soil community of Manao-Pee cave at (a) class, (b) order, (c) family, and (d) genus level by amplicon sequencing versus shotgun metagenomic sequencing. In general, at deeper taxonomic levels, shotgun metagenomic sequencing gave more insight into the numbers and identity of microbial present. (DOCX 12712 kb) [file 12866_2019_1521_MOESM15_ESM.docx]

**Additional file 15: Figure S6.** Comparison of bacterial diversity in the soil community of Manao-Pee cave at (a) class, (b) order, (c) family, and (d) genus level by amplicon sequencing versus shotgun metagenomic sequencing. In general, at deeper taxonomic levels, shotgun metagenomic sequencing gave more insight into the numbers and identity of microbial present.

**a**


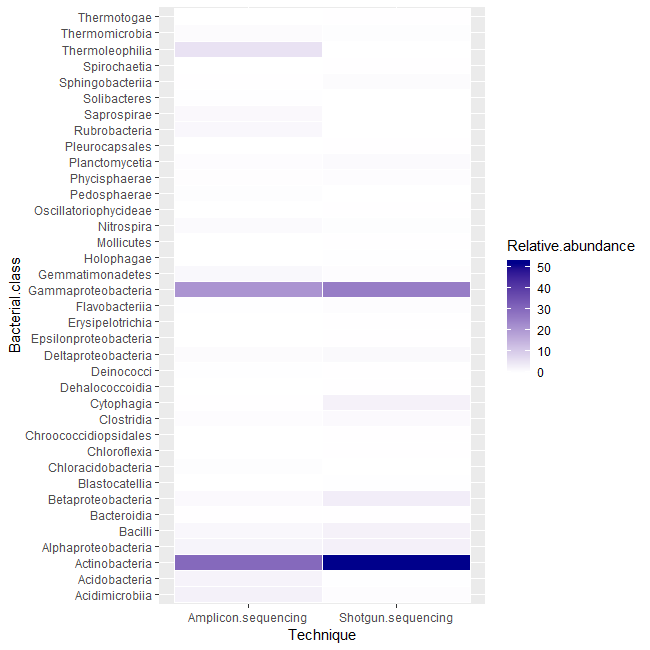


(%)


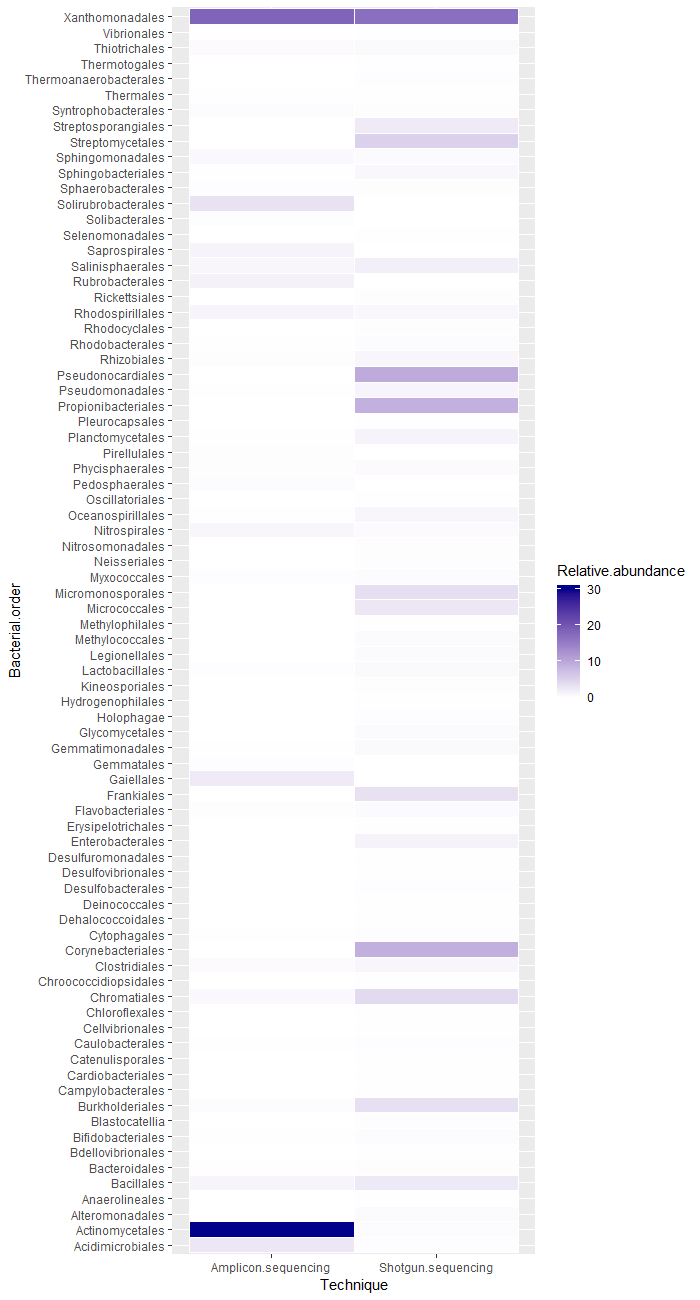


(%)

**b**


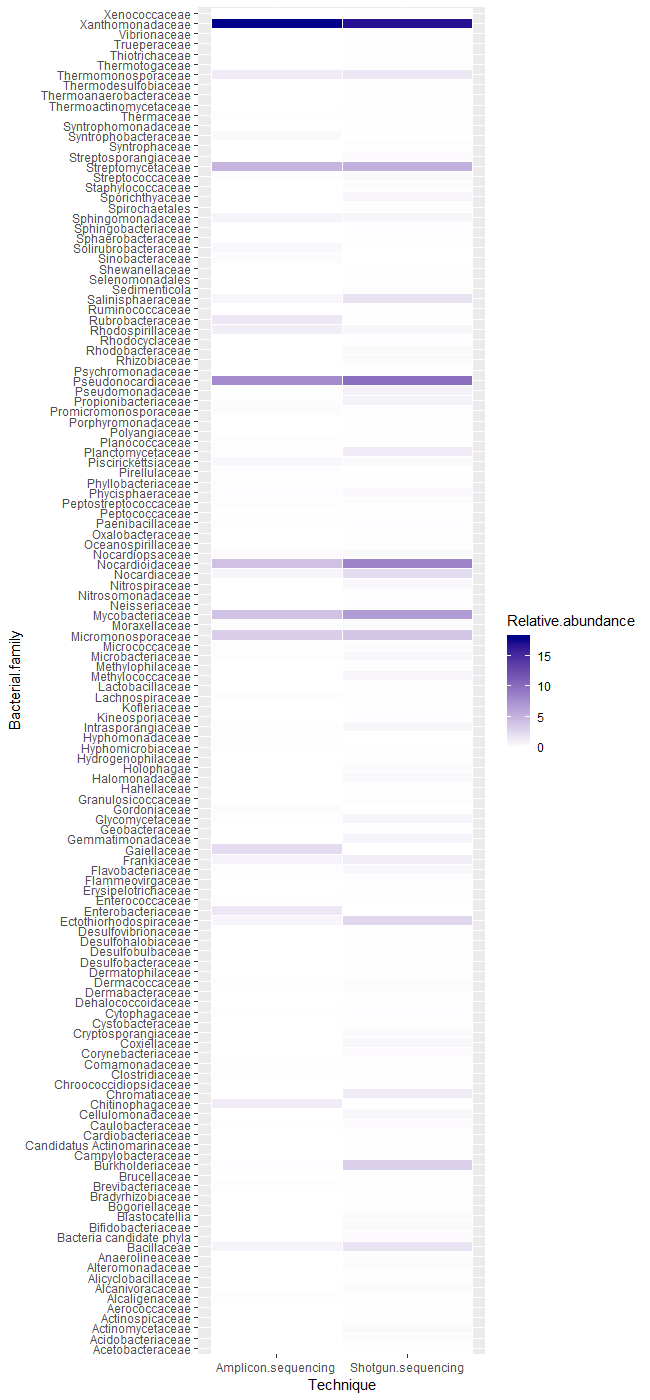


(%)

**c**


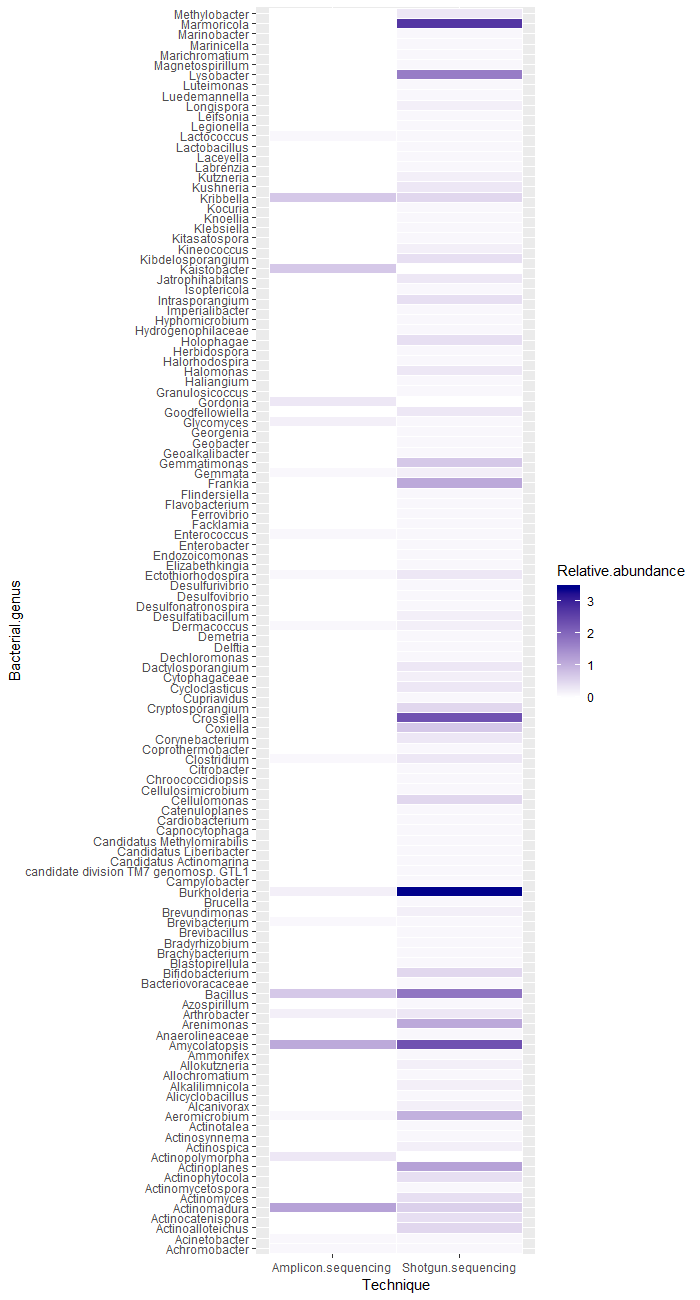


(%)

**d**


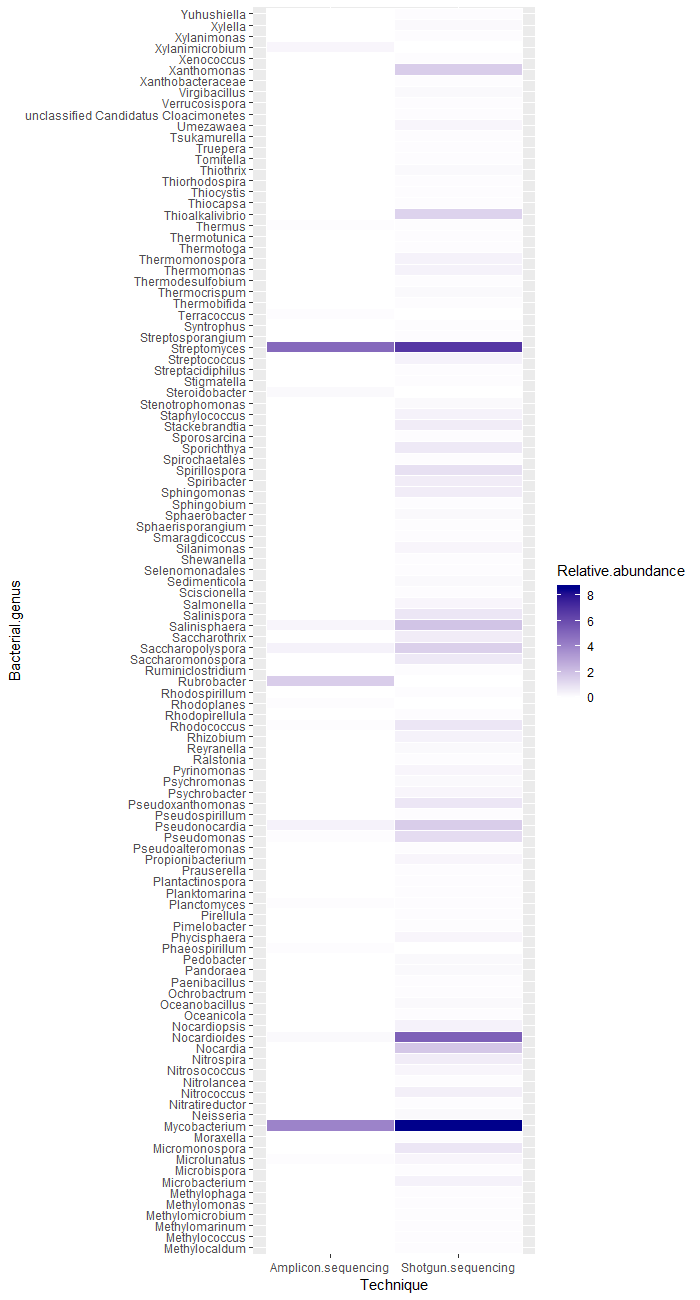


(%)

**d**
